# CHD1 Contributes to Intestinal Resistance against Infection by *P. aeruginosa* in *Drosophila melanogaster*

**DOI:** 10.1371/journal.pone.0043144

**Published:** 2012-08-13

**Authors:** Johanna Sebald, Stefano Morettini, Valerie Podhraski, Cornelia Lass-Flörl, Alexandra Lusser

**Affiliations:** 1 Division of Molecular Biology, Biocenter, Innsbruck Medical University, Innsbruck, Austria; 2 Division of Hygiene and Medical Microbiology, Innsbruck Medical University, Innsbruck, Austria; University College London, United Kingdom

## Abstract

*Drosophila* SNF2-type ATPase CHD1 catalyzes the assembly and remodeling of nucleosomal arrays *in vitro* and is involved in H3.3 incorporation *in viin vivo* during early embryo development. Evidence for a role as transcriptional regulator comes from its colocalization with elongating RNA polymerase II as well as from studies of fly *Hsp70* transcription. Here we used microarray analysis to identify target genes of CHD1. We found a fraction of genes that were misregulated in *Chd1* mutants to be functionally linked to *Drosophila* immune and stress response. Infection experiments using different microbial species revealed defects in host defense in *Chd1*-deficient adults upon oral infection with *P. aeruginosa* but not upon septic injury, suggesting a so far unrecognized role for CHD1 in intestinal immunity. Further molecular analysis showed that gut-specific transcription of antimicrobial peptide genes was overactivated in the absence of infection in *Chd1* mutant flies. Moreover, microbial colonization of the intestine was elevated in *Chd1* mutants and oral infection resulted in strong enrichment of bacteria in the body cavity indicating increased microbial passage across intestinal epithelia. However, we did not detect enhanced epithelial damage or alterations of the intestinal stem cell population. Collectively, our data provide evidence that intestinal resistance against infection by *P. aeruginosa* in *Drosophila* is linked to maintaining proper balance of gut-microbe interactions and that the chromatin remodeler CHD1 is involved in regulating this aspect.

## Introduction

In contrast to most vertebrates, *Drosophila melanogaster* lacks an adaptive immune system, and host defense relies exclusively on various innate immunity mechanisms (reviewed e.g. in [1]–[5]). The activation of an immune response upon recognition of the invading microorganisms is controlled by a complex interplay of multiple signaling pathways that are widely conserved and serve similar roles in vertebrates. Two major signaling cascades regulate the production of antimicrobial peptide genes and other immune response reactions in the fly: the Toll and the immune deficiency (Imd) pathway. Both signal to transcription factors of the nuclear factor κB (NFκB)/Rel family, which are termed Dorsal, Dif and Relish [6].

The molecular mechanisms of *Drosophila* immune response have been studied in considerable detail in recent years, and various transcription factors beside the NFκB-like factors (e.g. dGATA, Caudal, Drifter, dAP1) are known to regulate the expression of immunity-related genes [3]. In contrast, relatively few studies have addressed the role of chromatin-based regulatory mechanisms for immunity-related genes in *Drosophila*. Modulation of chromatin structure and dynamics by posttranslational modification of histones, incorporation of variant histones or the action of ATP-dependent chromatin remodeling factors is a well-studied general mechanism in gene regulation (e.g. [7]–[10]). In vertebrates, histone modifications and the activity of SNF2-family chromatin remodeling factors have been demonstrated to play crucial roles in the regulation of immunity-related genes, such as NFκB target genes or other inflammatory response genes [11], [12].

The SNF2 family of proteins comprises a large group of ATP-utilizing motor proteins, the majority of which has chromatin-related functions (e.g. [13]). Of the 15 predicted subfamilies that were identified in *Drosophila* [14], only a handful has been studied in greater detail and even fewer with respect to their role in the immune response of the fly. Two prominent chromatin remodeling factors that have been found to be involved in the regulation of *Drosophila* defense mechanisms are the nucleosome remodeling factor (NURF) complex and Domino [15]–[17]. NURF, which contains the motor subunit ISWI, was demonstrated to act as a corepressor of STAT target genes, thereby modulating the JAK/STAT-mediated immune response [15], [18], [19]. Domino (Dom), a fly homolog of the yeast and mammalian Swr1 ATPases, has originally been described as a factor required for hemocyte formation [16], [20], [21] and was recently found to control the regulation of a large subset of immunity-related genes [17].

The chromatin remodeling factor chromo helicase domain protein 1 (CHD1) has been implicated in the regulation of transcription, in particular elongation. For example, CHD1 has been found to colocalize with the elongating form of RNA polymerase II, to interact with various elongation and mRNA processing factors and to affect the transcription of many genes in yeast and embryonic stem cells [22]. In *Drosophila*, we and others have found that CHD1 is required for full transcriptional induction of heat shock genes [23], [24]. In addition to its transcription-dependent functions, we have shown previously that CHD1 acts as a chromatin assembly and remodeling factor *in vitro* and that it is required for the transcription-independent incorporation of the histone H3 variant H3.3 during the reorganization of paternal pronuclear chromatin at fertilization *in vivo* [25], [26].

In an effort to further dissect the biological functions of CHD1 in *Drosophila*, we have performed gene expression profiling of *Chd1* wild-type and mutant larvae. We found, that a considerable fraction of genes that are misregulated in the absence of CHD1 are genes involved in *Drosophila* immune response, stress response and detoxification processes. Since CHD1 has not been previously linked to function in immunity-related mechanisms in any organism, we decided to more directly investigate this potential new role of CHD1. We observed that loss of CHD1 rendered flies susceptible to infection by the gram-negative bacterium *Pseudomonas aeruginosa* upon ingestion of the bacteria but did not affect sensitivity upon septic injury. We found that in *Chd1* mutants intestinal AMP levels and, at the same time, bacterial load of the gut were significantly elevated. Moreover, we show that guts of *Chd1* mutant flies allowed the passage of large numbers of bacteria into the fly body upon challenge with *P. aeruginosa*, which ultimately may be the cause of the flies’ death. Thus, we propose that CHD1 should be considered as a novel player contributing to intestinal resistance against microbial assault.

## Results

### Deletion of *Chd1* Leads to Misregulation of Immunity-linked Genes in *Drosophila* Larvae

**Figure 1:**
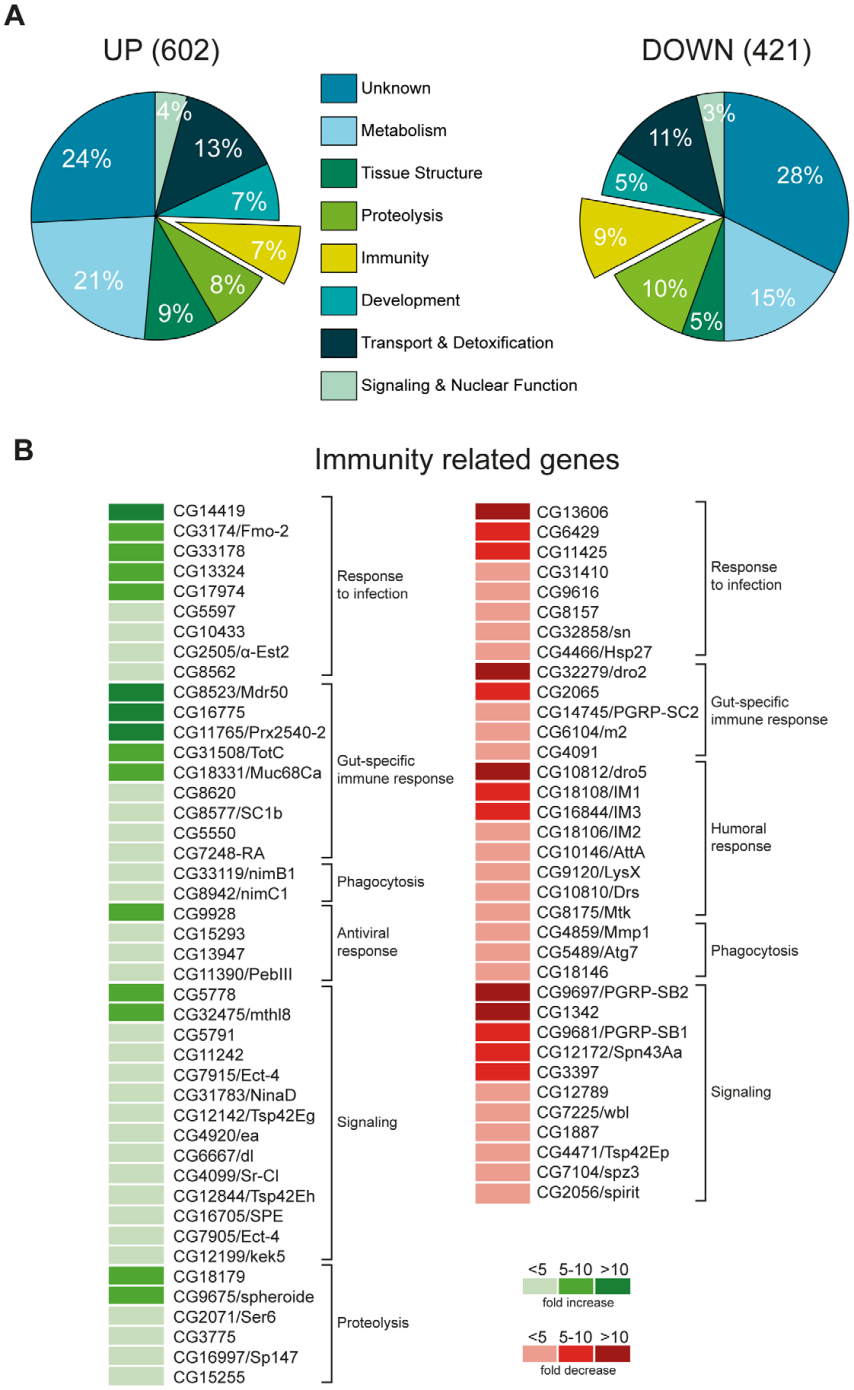
Whole genome expression profiling of *Chd1^−/−^* larvae. (A) Gene ontology classification of genes that display at least 2-fold up (left) or downregulation (right). Immunity-linked genes account for a considerable fraction of all misregulated genes. (B) Assignment of immunity-linked misregulated genes in *Chd1^−/−^* larvae to several functional subcategories. Color bars denote the magnitude of aberrant regulation.

In a search for novel functions of CHD1 we performed microarray analysis with RNA from *Chd1*-deficient (*Chd1^−/−^*; [26]) third instar larvae. To minimize genetic background effects, we used a line bearing a wild-type *Chd1* transgene in the *Chd1*-deficient background (hereafter termed *Chd1^WT/WT^*; [23]) to serve as the wild-type reference. We have previously shown that the expression levels of transgenic *Chd1* in this line equal those of *w^1118^* wild type flies [23]. Expression data were generated using Affymetrix GeneChip *Drosophila* Genome 2.0 arrays. Our analysis revealed that 602 genes were upregulated and 421 genes were downregulated at least 2-fold in *Chd1^−/−^* larvae (Tables S1 and S2). Subjecting the data to gene ontology analysis we found that a large portion of misregulated genes are linked to functions in metabolism, transport, detoxification and proteolysis. Interestingly, about 7% of upregulated and 9% of downregulated genes have assigned immunity-related functions (Figure 1A and Tables S1 and S2). Thus, in all at least 28% of the upregulated and 30% of the downregulated genes in *Chd1* mutant flies belong to pathways that are involved in stress response in a wider sense.

Nothing has been known so far about a role of the CHD1 remodeler in immunity-linked processes. Therefore, we set out to more closely study this possibility. In a first step, we further analyzed the group of immunity-linked genes with regard to functional subcategories (Figure 1B, Tables S1 and S2). We found that various components of immunity-related signaling cascades were misregulated in the absence of CHD1. Examples are SPE (spätzle-processing enzyme), Spheroide and Easter, which are proteases involved in activating the Toll ligand Spätzle [1], [27] or the Toll pathway-specific transcription factor Dorsal, all of which showed higher expression levels in *Chd^−/−^* larvae. Several effector genes of the humoral response, which are targets of immunity-related signaling cascades, were downregulated with some of them showing exceptionally strong reduction in transcript levels (e.g. *drosomycin 2,* 50-fold; *dro5,* 11-fold; *immune induced molecule 1,* 10-fold; *IM3,* 7-fold). Strongly decreased transcription was also observed for the genes encoding the pattern recognition proteins PGRP-SB1/2 and PGRP-SC2, which are thought to negatively regulate host defense response [28]–[30]. Various up- and downregulated genes show gut specific expression patterns (flybase.org) or have reported relevance for gut immunity. For example, two very strongly activated genes, *CG16775* and *CG11765*, are predominantly expressed in the larval gut, and they have been found to undergo gene expression changes upon bacterial infection [31], [32]. Other genes in this group include *CG31508*, encoding a small protein with no characterized function, that had previously been identified to be strongly induced in the gut upon infection in a Rel-dependent manner [33], a fibrinogen-related protein gene (*CG5550*), a gene encoding a potential constituent of the gut peritrophic matrix (*CG7248*) and a peptidoglycan recognition protein gene (*PGRP-SC1b*), which acts as a negative regulator of immunity-regulating signal transduction pathways [29]. All of these genes have been related to infection-induced transcriptional misregulation [31], [34]. Other gut-specific genes that were downregulated were *dro2* and *PGRP-SC2*. Furthermore, *CG6104*, a gene of the *E(spl)* region that has been implicated in gut stem cell maintenance [35] and *CG4091*, a gene encoding a putative caspase inhibitor that shows high expression levels in the larval midgut and was linked to autophagic cell death [36], were downregulated in *Chd1^−/−^* larvae.

### CHD1 Mutants are Susceptible to Oral Infection by the Gram-negative Bacterium *P. aeruginosa*

**Figure 2:**
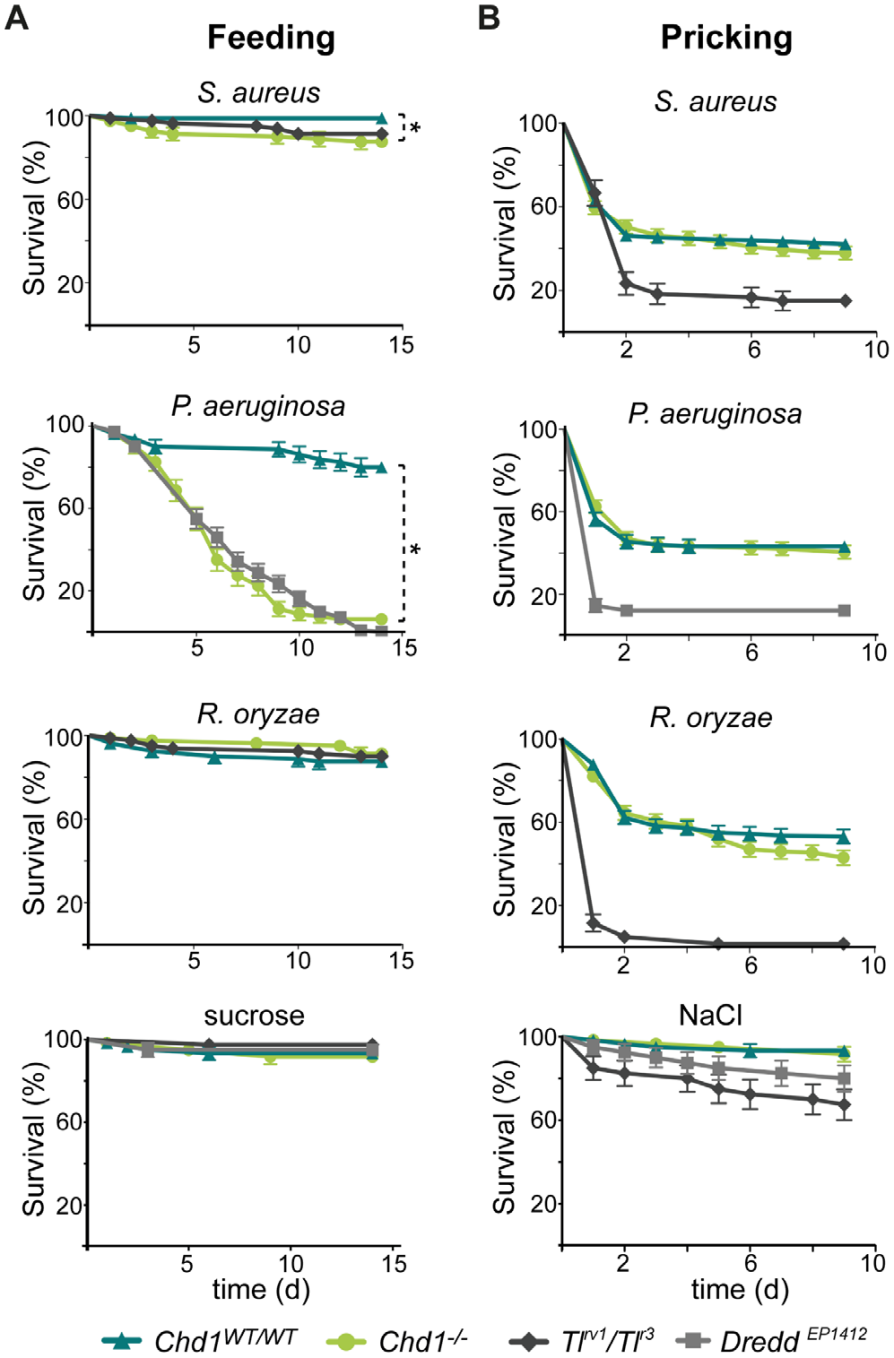
Loss of CHD1 renders flies more susceptible to oral but not to systemic infection by *P. aeruginosa*. (A) Kaplan-Meier plots displaying survival rates of female *Chd1^−/−^*, *Chd1^WT/WT^*, *Tl^rv1^/Tl^r3^* and *Dredd^EP1412^* flies after feeding with 5% sucrose solution containing either *S. aureus*, *P. aeruginosa* (both 10^9^–10^10^ cfu/ml), *R. oryzae* (5×10^8^ spores/ml) or no microbes for 15 h. *Chd1-*deficient flies are significantly more susceptible to oral infections with *S. aureus* and *P. aeruginosa* (*P<0.05; n = 80) than *Chd1^WT/WT^* flies. (B) Survival rates of female *Chd1^−/−^*, *Chd1^WT/WT^*, *Tl^rv1^/Tl^r3^* and *Dredd^EP1412^* flies following septic injury with different microbe solutions or NaCl as above (10^6^–10^7^ cfu or spores/ml). No significant differences of survival of infected *Chd1^−/−^* (n = 200) and *Chd1^WT/WT^* (n = 320) flies were observed.

In light of the fact that various immune response and stress response pathways were misregulated in *Chd1*-deficient larvae, we next sought to investigate, whether this aberrant transcription program affects the ability of the flies to combat microbial infections. In nature, infection of *Drosophila* typically occurs through the entry of microbes via ingestion or via the respiratory system eliciting a local immune response mediated by the epithelia. Thus, to study if CHD1 affects defense mechanisms in the intestine, flies were fed with sucrose solution containing either the gram-positive bacterium *Staphylococcus aureus*, the gram-negative *Pseudomonas aeruginosa* or the fungus *Rhizopus oryzae*. These experiments revealed that the absence of CHD1 severely hampered survival of the flies after infection with *P. aeruginosa*. About 50% of *Chd1*-mutant flies had succumbed to the infection after 5 days, and less than 5% were alive after 14 days compared to 80% of the *Chd1^WT/WT^* line (Figure 2A). Thus, *Chd1*-mutant flies show similar susceptibility to *Pseudomonas* infection as the Imd-pathway mutant *Dredd^EP1412^* (Fig. 2A). In contrast, only a small, albeit statistically significant, decrease in viability of *Chd1^−/−^* flies was observed after infection with *S. aureus*, and no differences to the wild type became apparent when *R. oryzae* spores had been ingested by the flies (Figure 2A). These results show that CHD1 is indeed involved in host defense mechanisms, in particular the local response to *P. aeruginosa* infection.

### CHD1 is not Required for Fighting Systemic Infections in *Drosophila*

When microbes are able to escape the first line of *Drosophila* immune response that consists of local production of AMPs and reactive oxygen species (ROS) by the epithelia, or if they breach the epithelial barrier in case of wounding, a systemic immune response is elicited [1]. Since we have observed that the absence of CHD1 renders flies more susceptible to oral infection, we determined whether CHD1 is also required for the defense against systemic infection. To this end, we infected *Chd1*-deficient and control flies with *S. aureus*, *P. aeruginosa* and *R. oryzae* by septic injury. Interestingly, these experiments did not reveal evidence that *Chd1^−/−^* flies were more sensitive towards infection than the rescued line (Figure 2B). Conversely, the Toll-mutant *Tl^rv1^/Tl^r3^* line [37] showed a clear decrease in viability upon infection with *S. aureus* or *R. oryzae,* and *Dredd^EP1412^* flies were highly susceptible to *P. aeruginosa* (Figure 2B). These findings are consistent with the notion that the Toll pathway predominantly acts in response to gram-positive bacteria and fungi and that the Imd pathway is necessary to combat infection by gram-negative bacteria [1].

Thus, in sharp contrast to oral infection, where loss of *Chd1* renders flies susceptible to infection, CHD1 does not appear to impact on the systemic immune response.

### Analysis of the Cellular Immune Response in *Chd1*-mutant Larvae

Due to the fact that our microarray analysis data indicated the misregulation of a number of genes linked to cellular host defense, such as *Nimrod* and *Tetraspanin* receptor genes (Fig. 1B, Tables S1, S2), we examined, whether increased susceptibility to infection was related to impaired hemocyte function. We found that hemocyte numbers as well as their ability for phagocytosis (as tested by injection of india ink into the larval hemocoel [38]) were similar in *Chd1*-mutant and –rescued larvae (Fig. S1A, B). Likewise, wound healing-coupled melanization proceeded without difference in wild-type and mutant larvae after injury of third instar larvae by pricking with a sterile needle (Fig. S1C). Thus, these results suggest that increased susceptibility of *Chd1*-mutant flies is likely not due to a defective cellular immune response.

### AMP Genes are Derepressed in *Chd1*-mutant Flies

**Figure 3:**
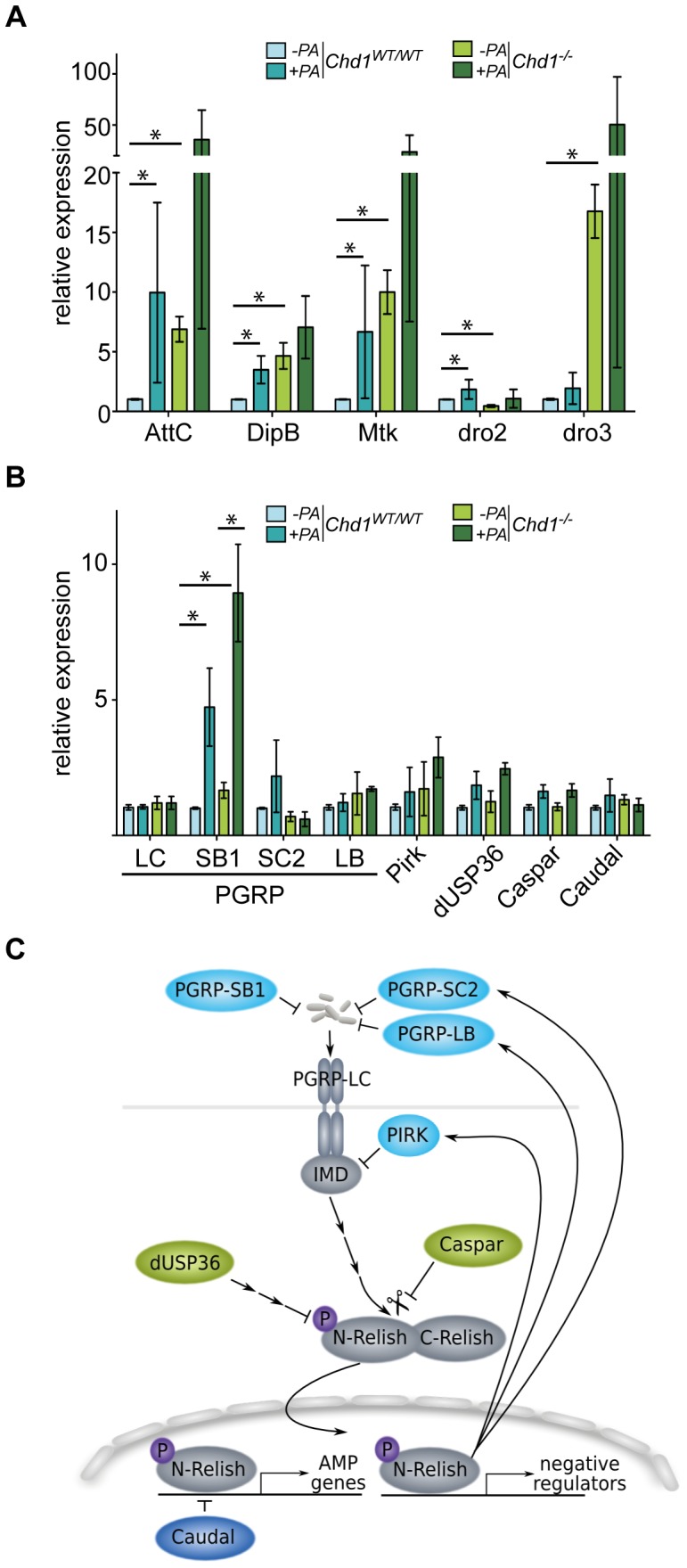
CHD1 affects the expression of AMP genes in the gut. (A) Expression of several AMP genes is significantly upregulated in *Chd1^−/−^* flies in the absence of infection. (B) The expression of several regulators of Imd pathway activity is not significantly altered in *Chd1^−/−^* flies. RT-qPCR analysis of isolated guts of unchallenged (*−PA*) and *P. aeruginosa* infected (*PA*; 15 h) *Chd1^−/−^* and *Chd1^WT/WT^* flies was performed. Transcript levels of indicated genes were normalized against *Rpl32* and are expressed relative to those of the respective gene in *Chd1^WT/WT^* guts. Values represent mean +/− SD of at least 3 independent experiments with 50 guts each (*P<0.05).

To investigate the molecular mechanisms of the contribution of CHD1 to intestinal immunity, we measured expression levels of different AMP genes by RT-qPCR in guts prepared from adult *Chd1^−/−^* and *Chd1^WT/WT^* flies. We analyzed mRNA levels of *Attacin C* (*AttC*) and *Diptericin B* (*DipB*), which are target genes of the Imd signaling pathway, and *Metchnikowin* (*Mtk*), which is known as a Toll-regulated gene in systemic immune response but is controlled by the Imd signaling pathway as well as by the transcription factor dGATAe in the gut [33], [39], [40]. Surprisingly, expression of *AttC*, *DipB* and *Mtk* was significantly higher in intestines from *Chd1* mutant flies than in those from control flies (Figure 3A). We further tested the expression of the AMP genes *Drosomycin 2* (*dro2*) and *Drosomycin 3* (*dro3*), the latter of which had been shown to be controlled by the JAK/STAT pathway [33]. Similar to the other AMP genes, *dro3* transcription was strongly overactivated in guts from *Chd1^−/−^* flies (Figure 3A). In contrast, *dro2* mRNA levels were decreased in the absence of CHD1 (Figure 3A). Together these data indicate a latent activation of intestinal immune response in *Chd1* mutant flies even in the absence of bacterial challenge.

### Expression of AMP Genes upon Infection

Next we determined whether CHD1 is required for the regulation of AMP gene expression upon infection. We isolated RNA from guts of flies after 15 h of infection with *P. aeruginosa* and measured the expression of the selected AMP genes by RT-qPCR. We found that in the *Chd1^WT/WT^* flies, ingestion of *P. aeruginosa* elicited the activation of all tested AMP genes by various degrees ranging from ∼9-fold (*AttC*) to ∼2-fold induction (*dro2*; Figure 3A). In contrast, none of the tested AMPs showed statistically significant levels of induction in *Chd1^−/−^* flies upon bacterial ingestion (Figure 3A). Note, that variations in AMP expression levels upon infection were rather pronounced across different experiments. Although these experiments were performed up to 6 times with 50 guts each, this problem could not be solved. It is possible that different feeding behavior of the flies or subtle differences in the inoculum are responsible for this effect. However, because in some experiments AMP expression was clearly induced (Figure 3A), we conclude that, in principle, AMP activation is still possible in the mutant flies and therefore not dependent on CHD1.

Together these results indicate that CHD1 most likely does not impact on the induction of AMP expression in response to bacterial challenge, but that it contributes to the maintenance of proper AMP levels in the absence of infection. One possible way by which the chromatin remodeler CHD1 might affect this process is by directly or indirectly regulating transcriptional read-out of immune response signaling pathways, in particular of the Imd (e.g. *AttC, DipB, Mtk*) and the JAK/STAT (e.g. *dro3*) pathways.

### AMP Overactivation in *Chd1* Mutant Guts is not Due to a Misregulation of Immunosuppressive Genes

To examine, whether the elevated steady state AMP levels in *Chd1* mutant guts are due to defects in the regulation of the intestinal Imd immune signaling pathway, we analyzed the expression levels of a number of genes that are known for their roles in this process. Beside the repressors of basal AMP transcription Caudal (Cad; [41]), Caspar [42] and dUSP36 [43], we tested transcript levels of *Pirk/PIMS/Rudra*, which mediates negative feedback regulation of Imd signaling [44]–[46], as well as of four members of the PGRP family (SB1, SC2, LB, LC; Fig. 3C). PGRP-LC is a membrane receptor involved in sensing gram-negative bacteria [47]–[49], PGRB-LB and –SC2 have been described as negative regulators of Imd pathway activity [30], [50], whereas no obvious functions in the immune response have been detected so far for PGRP-SB1 [28]. None of the negative regulator genes *PGRP-LB, PGRP-SC2, Pirk, caspar, caudal* and *dUSP36* or the positively acting receptor *PGRP-LC* showed altered expression in *Chd1*-mutant compared to wild-type guts (Figure 3B). *PGRP-SB1* transcript levels were slightly elevated in the mutant in the absence of bacterial challenge and strongly induced upon infection (Figure 3B). *PGRP-SB1* has been shown to be induced in a Rel-dependent way by bacterial infection [33]. Therefore, its upregulation in the *Chd1* mutant is consistent with the observed derepression of other Imd target genes, such as *AttC*, *DipB* and *Mtk* (Figure 3A). However, these data do not point to a role for CHD1 in interfering with the negative regulation of the Imd pathway.

### *Chd1^−/−^* Flies have Normal Numbers of Intestinal Stem Cells

**Figure 4:**
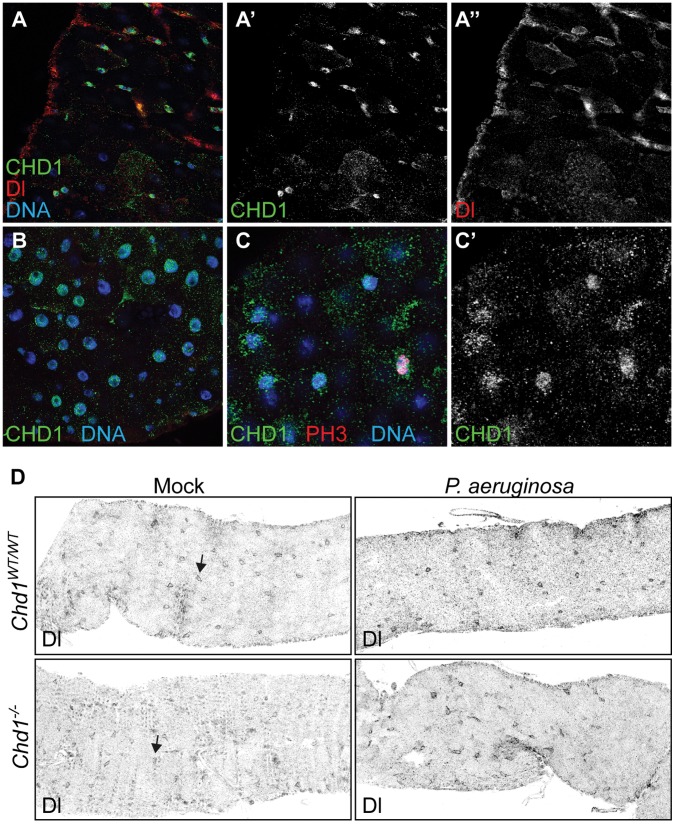
Deletion of *Chd1* does not cause aberrant numbers or distribution of ISCs in the fly intestine. (A, A’, A”) Co-staining of isolated guts of *Chd1^WT/WT^* flies with antibodies against CHD1 and Delta revealed localization of CHD1 to ISCs. (B) CHD1 is present in the nuclei of large enterocytes. (C, C’) CHD1 colocalizes with mitotic, PH3-positive cells. CHD1, green; Dl, red; PH3, red; DNA was visualized by staining with DAPI (blue). (D) Guts of unchallenged and *P. aeruginosa* infected *Chd1^−/−^* and *Chd1^WT/WT^* flies were stained with anti-DI antibody. An area of the anterior midgut is shown. Images are presented with inverted colors to enhance clarity. Arrows indicate individual ISCs with cell membrane-associated Dl signal. No significant differences with respect to number or distribution of ISCs was observed in uninfected and infected wild-type and mutant flies.

We next examined whether CHD1 plays a role not only in AMP production but also in gut cell homeostasis. Gut-specific immune response not only relies on Imd-mediated AMP expression but also on the production of ROS [1], [51]. A side effect of the release of ROS is the damage of epithelial cells. Gut cell renewal is orchestrated by a variety of signaling pathways, including the Notch, WNT, JAK/STAT, EGRF and p38 pathways [52]–[57]. As a consequence of their activity intestinal stem cell (ISC) division and differentiation of progenitor cells is induced [53], [58]. Misfunctioning of this replenishment cycle has been shown to gravely affect viability of the fly upon infection [54]. Using immunofluorescence microscopy we first examined CHD1 localization in guts from wild-type flies and found that CHD1 was present in all cell types, including ISCs and dividing cells, which were visualized by staining with antibodies against Delta (Dl) and phosphorylated histone H3 (PH3), respectively (Figure 4A–C). Because it was shown that mammalian CHD1 is required for the maintenance of pluripotency of mouse embryonic stem cells [59], we analyzed the number and distribution of Dl^+^ ISCs in intestines from *Chd1^−/−^* flies. These experiments revealed no obvious differences between *Chd1^−/−^* and *Chd1^WT/WT^* flies (Figure 4D). Similar results were obtained with guts from infected animals at 12 h, 2 and 4 days of infection (an exemplary image is shown in Figure 4D). Of note, the number of PH3-positive cells did not increase upon infection neither in wild-type nor in the mutant guts, indicating that no significant ISC proliferation was induced by the ingestion of *P. aeruginosa* in our system. Moreover, expression levels of the *escargot* (*esg*) gene, which is frequently used as a marker of stem cells and early differentiating cells, were not significantly altered in *Chd1^−/−^* versus *Chd1^WT/WT^* flies (data not shown).

Given that *Chd1*-mutant flies die with high frequency upon *P. aeruginosa* ingestion (Figure 2A), we would have expected enhanced ISC proliferation as a consequence of increased epithelial damage. Signaling from damaged cells has been shown to induce ISC proliferation [54]. To examine, if cell damage occurs upon infection in *Chd1^−/−^* guts, we performed immunostainings with antibodies against activated caspase 3 but were unable to detect increased cell death in the guts of *Chd1^−/−^* flies (data not shown). Note, that guts from control flies fed with SDS showed clear caspase 3 staining (Figure S2). Also, the overall appearance of intestines from non-infected or infected *Chd1*-mutant flies was similar (Figure S3). Thus, the strong susceptibility of *Chd1^−/−^* flies to oral infection by *P. aeruginosa* appears not to be due to major degeneration of gut epithelia. Together, these results indicate that cell renewal is not particularly stimulated under our infection conditions. Nevertheless, CHD1 appears to have no major role in the maintenance of the stem cell population in the gut.

### Increased Microbial Colonization of Guts from *Chd1^−/−^* Flies

**Figure 5:**
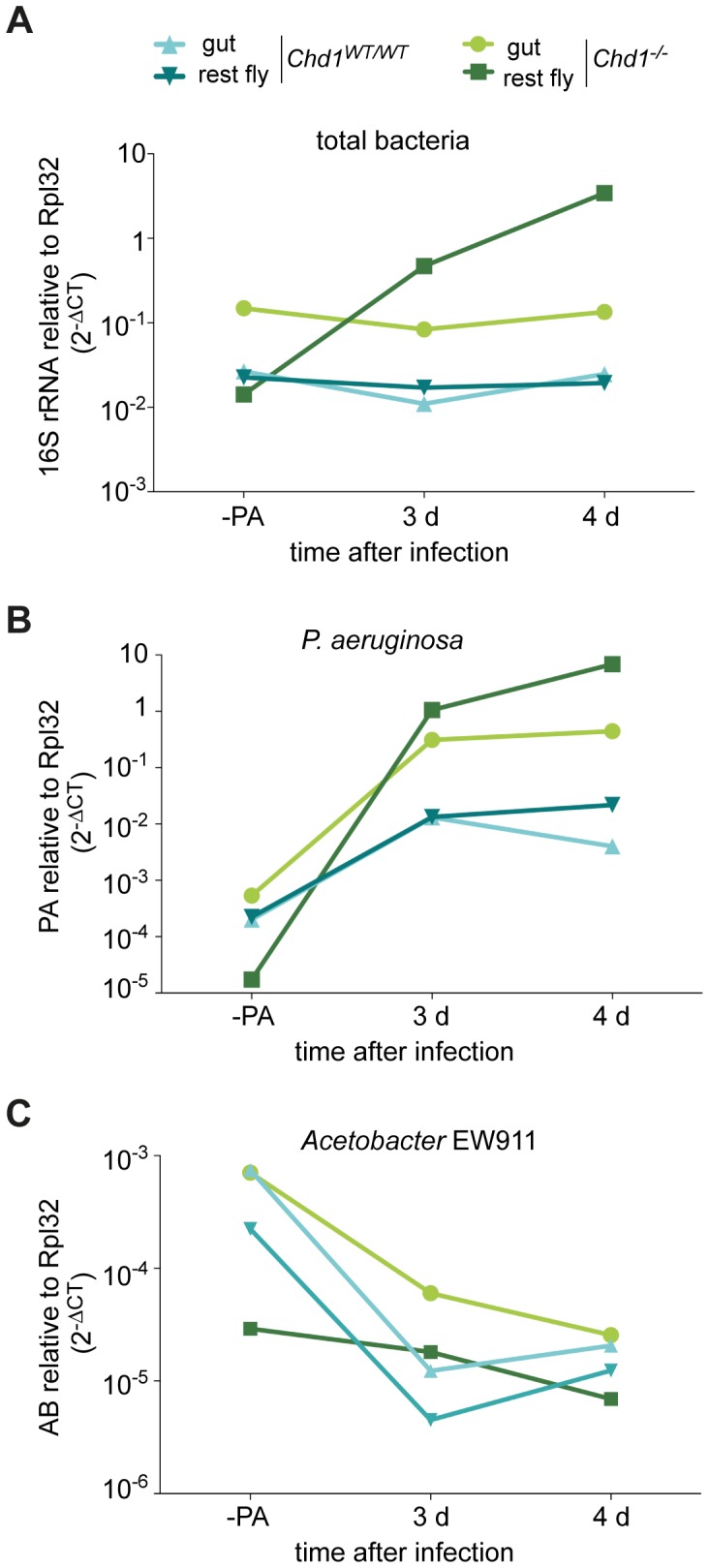
Bacterial load is elevated in *Chd1*-mutant flies. (A) Bacterial load was analyzed in isolated guts and in whole flies from which intestines had been removed. qPCR was performed with primers targeting 16S rDNA in the absence of infection (−PA) as well as 3 days and 4 days after oral infection with *P. aeruginosa.* (B) 251659264*P. aeruginosa* titers are strongly increased in *Chd1^−/−^* flies after infection. qPCR as in (A) with primers specific for *P. aeruginosa*. (C) Analysis of the gut-specific bacterium *Acetobacter* EW911. qPCR as in (A) with primers specific for *Acetobacter EW911*. Relative differences of bacterial genes and the fly *Rpl32* gene are expressed as 2^−ΔCT^ values. Values represent mean +/− SD of three independent experiments. Note that SD values are too small to show in the graph.

To further explore the causes for the increased mortality of *Chd1^−/−^* flies upon *P. aeruginosa* infection, we considered that elevated intestinal AMP levels, observed in the absence of CHD1, might affect the titers and/or community structure of commensal microbes. Such an effect has been observed before when the AMP-specific transcriptional repressor Cad was knocked down [41]. We examined the bacterial load in wild-type and mutant flies by qPCR. Interestingly, we found profoundly increased titers of bacteria in dissected guts from *Chd1^−/−^* flies in the absence of *P. aeruginosa* ingestion as well as after infection (Figure 5A and Figure S4A). To obtain evidence for potential alterations in the community structure of commensal bacteria in these densely colonized guts, we determined the relative abundance of two known gut-specific bacterial strains, *Acetobacteriaceae* strain EW911 and *Gluconobacter* sp. strain EW707 [41]. We found that in the absence of infection titers of *Acetobacter* EW911 were similar in wild-type and mutant flies (Figure 5C and Figure S4C). However, when taking into account the increase in overall bacterial titer in *Chd1^−/−^* intestines (Figure S4A), the proportion of *Acetobacter* EW911 within total microbiota actually is reduced (compare Figure S4A and C). The same results were obtained for *Gluconobacter* EW707 (Figure S5). Hence, the increased bacterial load in unchallenged guts in the absence of CHD1 appears not to be due to an even amplification of all bacteria but to be caused by an enrichment of (an) undefined species.

Analysis of the bacterial load in fly bodies from which the gut had been removed (“rest fly”) revealed that it was similar in wild type and mutant in the unchallenged state. In contrast, we observed a dramatic increase of bacteria in mutant, but not in wild-type flies, at 3 and 4 days of infection, respectively, indicating invasion of microbes from the gut into the body cavity (Figure 5A and Figure S4A). We then measured *P. aeruginosa* titers in the absence of infection and found no differences between *Chd1^−/−^* and *Chd1^WT/WT^* guts (Figure 5B). However, in *Chd1* mutant intestines *P. aeruginosa* showed 25 fold higher enrichment after 3 days of infection and ∼100 fold enrichment after 4 days of infection compared to wild-type guts (Figure 5B and Figure S4B). The accumulation of *P. aeruginosa* was even stronger in the body of mutant flies, where it increased to levels more than 300 fold higher than those of the wild-type flies (Figure 5B and Figure S4B). These data indicate that the intestinal environment in *Chd1* mutant flies allows ingested *P. aeruginosa* to accumulate to considerably higher numbers. Moreover, the dramatically increased bacterial titers in the fly body outside the gut after oral infection suggest that the gut epithelia is much more permissible to the passage of *P. aeruginosa* and possibly other bacteria into the hemolymph, which may ultimately result in the death of the fly.

## Discussion

### Misregulation of Humoral Response Genes in the Absence of CHD1

Our study shows that the chromatin remodeling factor CHD1 is involved in the regulation of immunity-related processes in *Drosophila melanogaster*. In particular, it appears to play an important part in gut-specific host defense against the gram-negative bacterium *P. aeruginosa*. We found that CHD1 affects the transcriptional regulation of AMP genes, which are under the control of the Imd as well as the JAK/STAT pathways. Intriguingly, gut-specific expression of *AttC*, *DipB*, *Mtk* and *dro3* was significantly overactivated in adult *Chd1^−/−^* mutant flies in the absence of infection. There are several explanations that may account for this phenomenon. First, CHD1 might function as a negative transcriptional regulator of AMP genes. Although overexpression of these AMPs was not evident from our microarray analysis of third instar larvae, we did detect significantly higher levels of *AttC*, *DipB* and *dro3* using RT-qPCR analysis of larval RNA (Figure S6). On the other hand, *Mtk* expression was downregulated in *Chd1^−/−^* larvae in contrast to adult guts, where it was overactivated (Figure S6 and Figure 3A). These findings may suggest that CHD1 is required for the proper regulation of immunity-associated genes in adults and in larvae. Our data from adult flies point to a corepressor function of CHD1. However, CHD1 has also been shown to act as a coactivator. For example, it is required in larvae to fully induce transcription of heat shock genes upon thermal stress [23]. Hence, CHD1 may act as both, a corepressor or coactivator of immune response genes depending on the developmental and/or tissue-specific context.

An alternative explanation for the observed upregulation of AMP levels in adult guts might be that CHD1 affects transcription of genes that are involved in the regulation of Imd pathway activity. Although we cannot formally rule out this possibility, our data showing wild-type-like levels of several such regulators (Cad, dUSP36, Casper, Pirk/PIMS/Rudra, PGRP-LB, LC, SC2, SB1) rather argue against it.

### Overactivation of AMP Genes - the Cause for Increased Infection Sensitivity of *Chd1^−/−^* Flies?

Alternatively or in addition to the explanations proposed above for the effect of *Chd1* deletion on AMP expression and fly survival upon infection, it is possible that CHD1 contributes to maintaining the fly intestine in a state that will allow for efficient combat of invading pathogens. It may be counterintuitive that guts, which express higher levels of AMPs, such as in *Chd1^−/−^* flies, should be more susceptible to infection by *P. aeruginosa* than those with normal AMP expression. Indeed, previous work has demonstrated that AMPs, in particular DipB, are critically involved in fighting oral infection by *P. entomophila* [60]. In our system, however, increased intestinal AMP levels caused by the absence of CHD1 did not confer resistance against *P. aeruginosa*. Although these results appear contradictory, they can be reconciled by considering the different degree of overexpression of *DipB* in both studies. While Liehl *et al.* [60] obtained strong overexpression of *DipB* using the UAS-Gal4 system (∼100 fold over uninfected state), *Chd1* mutation caused only about 2–4 fold higher *DipB* levels (Figure 3A). Thus, in our system, elevated AMP levels in *Chd1* mutants might not suffice to actively counteract *P. aeruginosa* infection. However, it is tempting to speculate that they may be adequate to exert continual selective pressure upon the commensal microbial community. As a result enrichment and overgrowth of less sensitive bacteria of the gut microbiota might occur, and these in turn may stimulate the expression of AMP genes, thus reinforcing the effects of *Chd1*-loss. This might weaken intestinal epithelia in a way to allow increased passage of *P. aeruginosa* into the body cavity. The facts that we find considerably elevated bacterial titers in the guts of *Chd1^−/−^* flies in the absence of infection (Figure 5A) as well as the strong enrichment of *P. aeruginosa* outside the gut upon infection (Figure 5B) are in good agreement with such a scenario. Of note, our experiments using septic injury did not show elevated susceptibility to *P. aeruginosa* infection of *Chd1^−/−^* compared to *Chd1^WT/WT^* flies. It may be possible that *P. aeruginosa* undergoes a switch in virulence when exposed to the altered bacterial colonization in the digestive tract of *Chd1*-mutant flies. For example, it has been shown before that coinfection of *Drosophila* with *P. aeruginosa* along with certain bacteria resulted in changed virulence factor gene expression and enhanced pathogenicity [61]. Alternatively and/or in addition, co-invasion of gut bacteria with *P. aeruginosa* into the hemolymph may cause increased lethality of the flies upon oral but not systemic infection.

### Role of CHD1 in Gut Cell Homeostasis

Lee and colleagues have recently shown that increased AMP production can indeed have adverse effects on the animal [41]. Overactivation of AMPs by inactivation of the transcriptional repressor Cad resulted in a disturbance of the commensal microbial community structure and consequently in epithelial damage and increased fly mortality [41]. Since epithelial damage is counteracted by the activity of ISCs and because it has been reported previously that mouse CHD1 is required for the maintenance of embryonic stem cell pluripotency [59], we determined, if ISC number or proliferation/differentiation was altered in *Chd1* mutants. In contrast to expectation, we observed normal numbers and distribution of ISCs in the absence of CHD1 arguing against an involvement of CHD1 in stem cell maintenance. In our experiments oral infection of either *Chd1^WT/WT^* or *Chd1^−/−^* flies with *P. aeruginosa* failed to cause gross morphological changes of the gut, induction of apoptosis in intestinal epithelia or ISC proliferation as was observed in other studies [55], [62]. Therefore, we cannot rule out that CHD1 plays a role in ISC proliferation or differentiation under different conditions.

The absence of gut tissue remodeling was also noted in a recent study, in which oral infection experiments were carried out with the *P. aeruginosa* P14 strain [63]. In this study it was concluded that the flies succumb to infection due to bacteremia rather than intestinal damage. Our data also suggest that oral infection of *Chd1*-mutant flies with *P. aeruginosa* leads to death by a mechanism that does not involve major gut degradation but instead correlates with increased bacterial load in the fly body.

Collectively, we have characterized a novel biological role for the chromatin remodeling and assembly factor CHD1 that is linked to immune response processes in *Drosophila*. Given the high degree of conservation of chromatin remodeling mechanisms across different species, it should be interesting to consider CHD1 also in studies of host defense in mammalian organisms.

## Materials and Methods

### Fly Strains

Flies were kept on standard cornmeal media at 25°C except for infection experiments, when flies were incubated at 29°C. *Chd1*-deficient (*Chd1^−/−^*) flies were obtained by crossing *Df(2L)Chd1^1^/CyO, GFP* with *Df(2L)Exel7014/CyO, GFP* [26], and *Chd1^WT/WT^* flies were obtained from crosses of *Df(2L)Chd1^1^,P{Chd1^WT^}/CyO, GFP* and *Df(2L)Exel7014, P{Chd1^WT^}/CyO, GFP* [23]. For *Tl*-deficient flies the temperature-sensitive *Tl^r3^* and *Tl^rv1^* alleles were combined [37]. *Dredd^EP1412^* flies [64] were obtained from the Bloomington Stock Center.

### Microbial Strains

*Rhizopus oryzae* AS82 (clinical isolate) was cultivated on potato-dextrose agar and spore suspensions were generated in 0.9% NaCl/0.01% Tween 80. Clinical isolates of *Pseudomonas aeruginosa* (998) and *Staphylococcus aureus* (877) were grown in tryptic soy broth at 37°C.

### Infection Experiments

Septic injury experiments were carried out by pricking the dorsal thorax of 2–4 day old female flies with a 27G needle that had been dipped into the respective microbe solution or 0.9% NaCl, respectively. The concentrations of the inocula were as follows: *R. oryzae*, 10^7^ spores/ml; *P. aeruginosa* and *S. aureus*, 10^6^ cfu/ml. Flies were incubated at 29°C and transferred to fresh food vials at every third day. For oral infection experiments bacterial pellets of overnight cultures were resuspended in 5% sucrose solution at an OD_600_ of 0.2–0.4 for *P. aeruginosa* and 1.8–2.0 for *S. aureus* and applied to Whatman filter discs covering the surface of a standard food vial. For fungal infection, food vials containing cornmeal glucose sucrose yeast extract agar for zygomycetes were inoculated with *R. oryzae* mycelia and fungal growth and sporulation was allowed for 3 days. 2–4 day old female flies were starved for 5 hours and then transferred to food vials containing contaminated filter discs/fungal mycelia or to filter discs soaked in sterile 5% sucrose solution for 15 h at 29°C. Subsequently, flies were transferred to fresh, uncontaminated vials and maintained at 29°C for 14 days with food changes at every third day. Dead flies were removed daily. Survival rates were calculated as percentage of living flies at each given time point. Data of 3–6 independent experiments with a minimum of 20 flies each were analyzed for statistical significance using the Kaplan-Meier log rank test (Prism 5.0 software).

### Microarray Analysis and Statistics

Total RNA was extracted from 3 independent pools of 40 wandering *Chd1^−/−^* and *Chd1^WT/WT^* 3rd instar larvae. Probe generation, hybridization to *Drosophila* Genome 2.0 arrays (Affymetrix) and data normalization was performed by the in-house Expression Profiling Unit (Innsbruck Medical University). Data analysis was performed using CARMAweb (Comprehensive R based Microarray Analysis web service) software package [65]. Microarray data were preprocessed using the gcRMA method and differentially expressed genes were identified in each of the three biological replicates using a fold change cut–off of 2. Statistical significance of differential regulation was determined by unpaired t-test analysis. To correct for multiple hypothesis testing problems the Benjamini and Hochberg adjustment method [66] was used. Functional annotation of the resulting gene lists was performed manually using information available on FlyBase (http://flybase.bio.indiana.edu).

### RT-qPCR

For gut-specific expression analysis, 50 guts (midgut plus hindgut) were dissected from female flies that were either uninfected or at 15 h after oral infection. Guts were frozen in liquid nitrogen immediately after dissection and RNA was prepared using the RNeasy Mini Kit (Qiagen). cDNA synthesis and RT-qPCR were performed as described [23]. qPCR primers are listed in Table S3. At least four independent experiments were performed for each condition and fly line. Statistical significance of differential regulation was determined using unpaired t-test analysis (Prism 5.0). The p-value for statistical significance was set at P<0.05.

### Determination of Bacterial Load

Twenty 2–4 day old female flies were washed with 70% ethanol, the complete intestinal tract was removed and the remaining carcasses were frozen in liquid nitrogen and subsequently homogenized in TES buffer (1 mM EDTA, 10 mM Tris-HCl pH 7.5, 100 mM NaCl) with a sterilized pestle. For gut-specific analysis female flies were washed with 70% ethanol and subsequently 10 guts (midgut plus hindgut) were dissected into sterile TES buffer. Genomic DNA was isolated by consecutive incubation of the samples with lysozyme (50 U/ml; 15 min, 37°C) and 1% SDS/2 mg/ml proteinase K (2 h, 37°C) with gentle shaking followed by phenol-chloroform extraction and ethanol precipitation. DNA pellets were dissolved in water and digested with RNase A (0.2 mg/ml). qPCR reactions with primers specific for bacterial 16S rDNA, *P. aeruginosa*, *Acetobacteriaceae* strain EW911 or *Gluconobacter* sp. strain EW707 (Table S3) were conducted in triplicate using a StepONEPlus instrument (Life Technologies) and Power SYBR Green PCR master mix (Life Technologies). The *Drosophila Rpl32* gene was used for normalization and 2^−ΔCT^ values were plotted.

### Immunocytochemistry and Microscopy

Guts of adult female flies were dissected into PBS and fixed for 15 min with methanol/heptane (1∶1). The guts were washed 3 times in 100% methanol, gradually transferred to PBST (PBS, 0.15% Triton X-100) and incubated with primary antibody diluted in 0.5%BSA/PBST overnight at 4°C. The following antibodies were used: PH3, Caspase 3 (both at 1∶250; Cell Signaling Technology), Delta (1∶50; Developmental Studies Hybridoma Bank) and CHD1 [67]. After secondary antibody (Alexa Fluor® 488 goat anti-rabbit IgG; Alexa Fluor® 594 goat anti-mouse IgG; Invitrogen) incubation, specimen were stained with DAPI and mounted in Vectashield (Vector Laboratories Inc.). Images were taken on a confocal laser scanning microscope (SP5, Leica). Images were processed using LSM Image Browser (version 4.2) software and Adobe Photoshop CS3.

### Hemocyte Count, Phagocytosis and Melanization Tests

Hemocytes were collected from 14 wandering 3^rd^ instar larvae and counted as described in [68]. Statistical differences were determined using unpaired t-test analysis (Prism 5.0). Phagocytosis was performed by injection of india ink into the body cavity of 3^rd^ instar larvae essentially as described by [38], except that a 30G 1/2” needle was used for injections. Melanization capability was tested as follows: 3rd instar larvae were washed in PBS and immobilized on double-sided tape attached to a glass slide. Sterile wounding was performed by pricking the larvae with a 30G 1/2” needle at abdominal segment A4 or A5. After wounding, larvae were transferred to a fresh glass slide and melanization was monitored every 10 min for 1.5 hours.

## Supporting Information

Figure S1 **Hemocyte function appears unaffected in** ***Chd1*** **-mutant larvae.** (A) Hemocyte numbers are similar in *Chd1^WT/WT^* and *Chd1^−/−^* larvae. Hemocytes were collected from larvae and counted in a hemocytometer. (B) Phagocytosis activity of mutant hemocytes is indistinguishable from that of wild-type hemocytes. White arrows indicate phagocytized ink particles in hemocytes. (C) Wound healing and melanization occurs in a similar fashion in wild-type and *Chd1*-mutant larvae. Black arrows indicate the site of wounding after 1.5 h.
(TIF)

Figure S2 **Detection of apoptosis by caspase 3 staining.** Guts from wild-type flies that were fed for 6 h with SDS or sucrose only were stained with antibodies against activated caspase 3 (green); DNA was visualized by DAPI staining (blue). A section of the anterior midgut is shown.
(TIF)

Figure S3 **Overall gut morphology is similar in** ***Chd1*** **-rescued and -mutant flies irrespective of the state of infection.** Several light microscopic images were taken along the anterior posterior axis of dissected guts (midgut plus hindgut) and subsequently assembled into one picture using Photoshop.
(TIF)

Figure S4 **Bacterial load is elevated in** ***Chd1*** **-mutant flies.** (A) Bacterial load was analyzed in isolated guts and in whole flies from which intestines had been removed. qPCR was performed with primers targeting 16S rDNA in the absence of infection (−PA) as well as 3 days and 4 days after oral infection with *P. aeruginosa.* (B) 251658240*P. aeruginosa* titers are strongly increased in *Chd1* ^−*/*−^ flies after infection. qPCR as in (A) with primers specific for *P. aeruginosa*. (C) Analysis of the gut-specific bacterium *Acetobacter* EW911. qPCR as in (A) with primers specific for *Acetobacter EW911*. The *Drosophila Rpl32* gene was used for normalization, and enrichment relative to the non-infected (−PA) *Chd1^WT/WT^* line was calculated using the 2^−ΔΔCT^ method. Values represent mean +/− SD of three independent experiments.
(TIF)

Figure S5 **Analysis of the gut-specific bacterium** ***Gluconobacter*** **EW707.** qPCR as in Figure S3 with primers specific for *Gluconobacter* EW707. (A) The relative differences of GB EW707 and the fly *Rpl32* gene are expressed as 2^−ΔCT^ values. (B) Enrichment relative to the non-infected (−PA) *Chd1^WT/WT^* line was calculated using the 2^−ΔΔCT^ method. Values represent mean +/− SD of three independent experiments.
(TIF)

Figure S6 **Several AMP genes are upregulated in** ***Chd1*** **^−^** ^***/*−**^ **larvae in the absence of infection.** Indicated AMP genes that did not score in the microarray were analysed by RT-qPCR. Transcript levels were normalized against *Rpl32* and are expressed relative to values obtained in *Chd1^WT/WT^* larvae. Values represent mean +/− SD of three independent experiments. Statistical significance was determined using unpaired t-test analysis (*P<0.05).
(TIF)

Table S1 **Upregulated genes in Chd1−/− flies.**
(PDF)

Table S2 **Downregulated genes in** ***Chd1^−^/^−^*** **flies.**
(PDF)

Table S3 **PCR Primer sequences.**
(PDF)
