# Supplementary figures and images for: CHD1 Contributes to Intestinal Resistance against Infection by P. aeruginosa in Drosophila melanogaster
Source: PLoS One. 2012 Aug 13;7(8):e43144. doi: 10.1371/journal.pone.0043144 (PMC3418260; doi:10.1371/journal.pone.0043144)

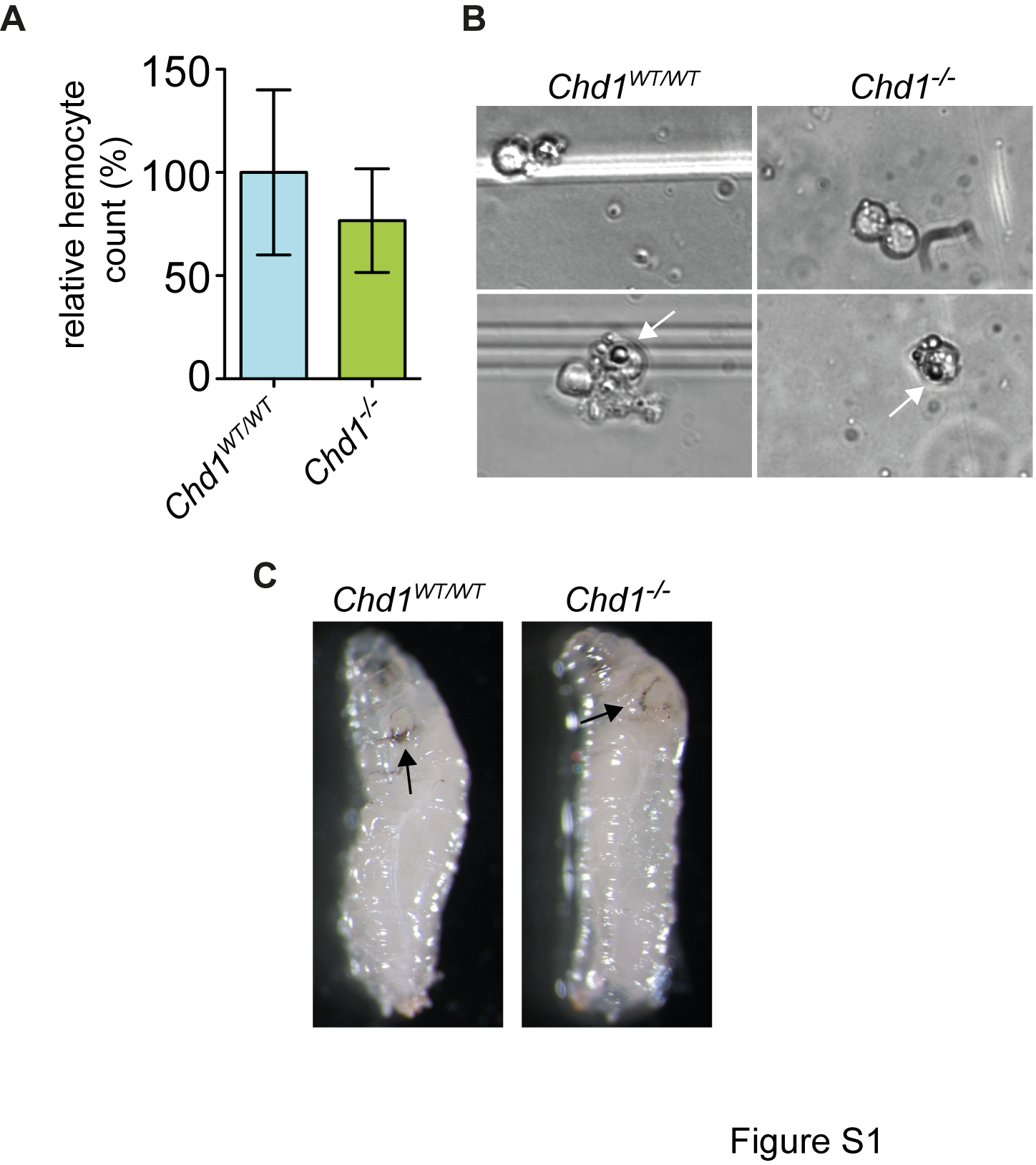

Supplement: Figure S1 — Hemocyte function appears unaffected in Chd1 -mutant larvae. (A) Hemocyte numbers are similar in Chd1WT/WT and Chd1−/− larvae. Hemocytes were collected from larvae and counted in a hemocytometer. (B) Phagocytosis activity of mutant hemocytes is indistinguishable from that of wild-type hemocytes. White arrows indicate phagocytized ink particles in hemocytes. (C) Wound healing and melanization occurs in a similar fashion in wild-type and Chd1-mutant larvae. Black arrows indicate the site of wounding after 1.5 h. (TIF) [file pone.0043144.s001.tif]

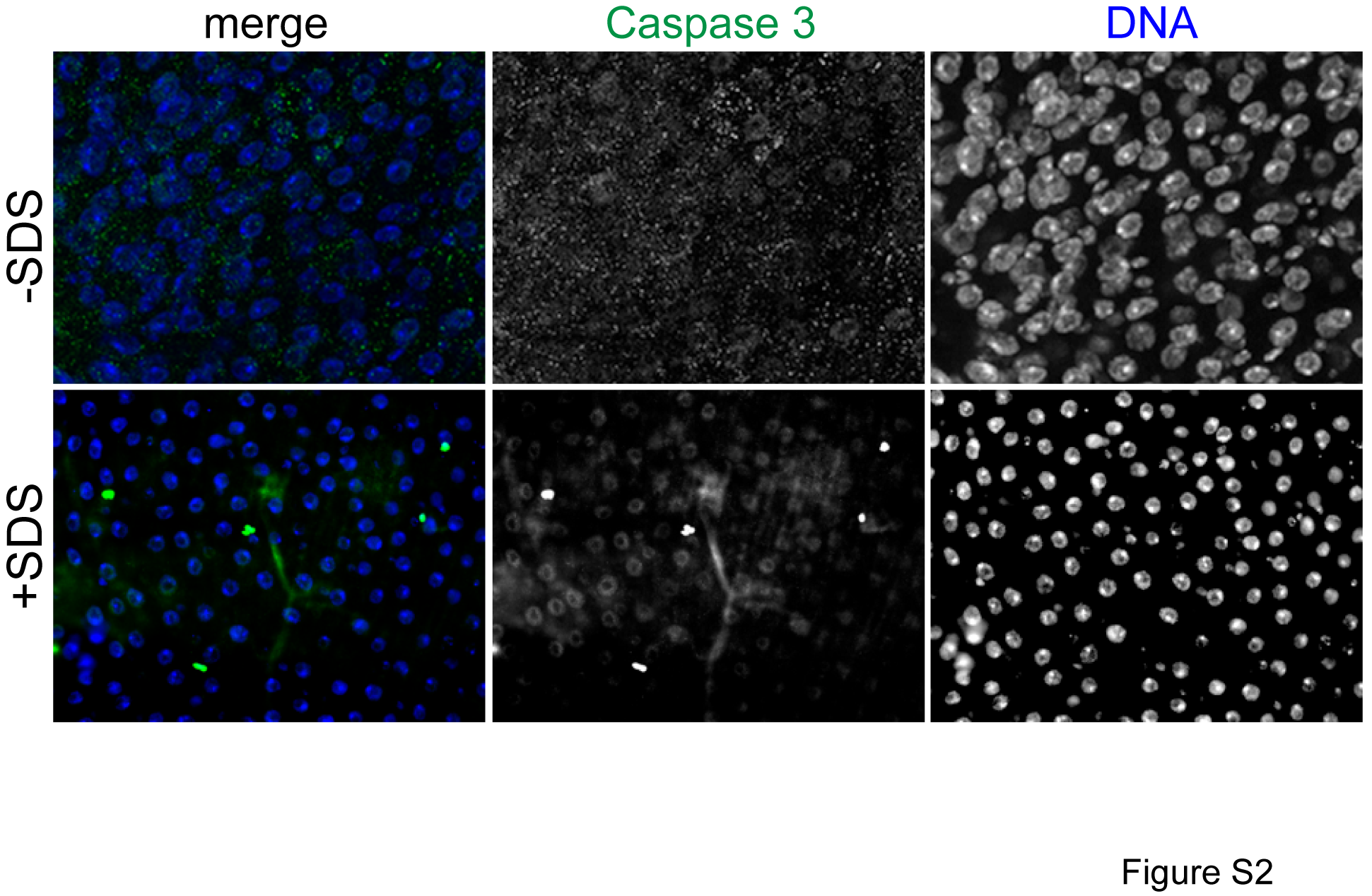

Supplement: Figure S2 — Detection of apoptosis by caspase 3 staining. Guts from wild-type flies that were fed for 6 h with SDS or sucrose only were stained with antibodies against activated caspase 3 (green); DNA was visualized by DAPI staining (blue). A section of the anterior midgut is shown. (TIF) [file pone.0043144.s002.tif]

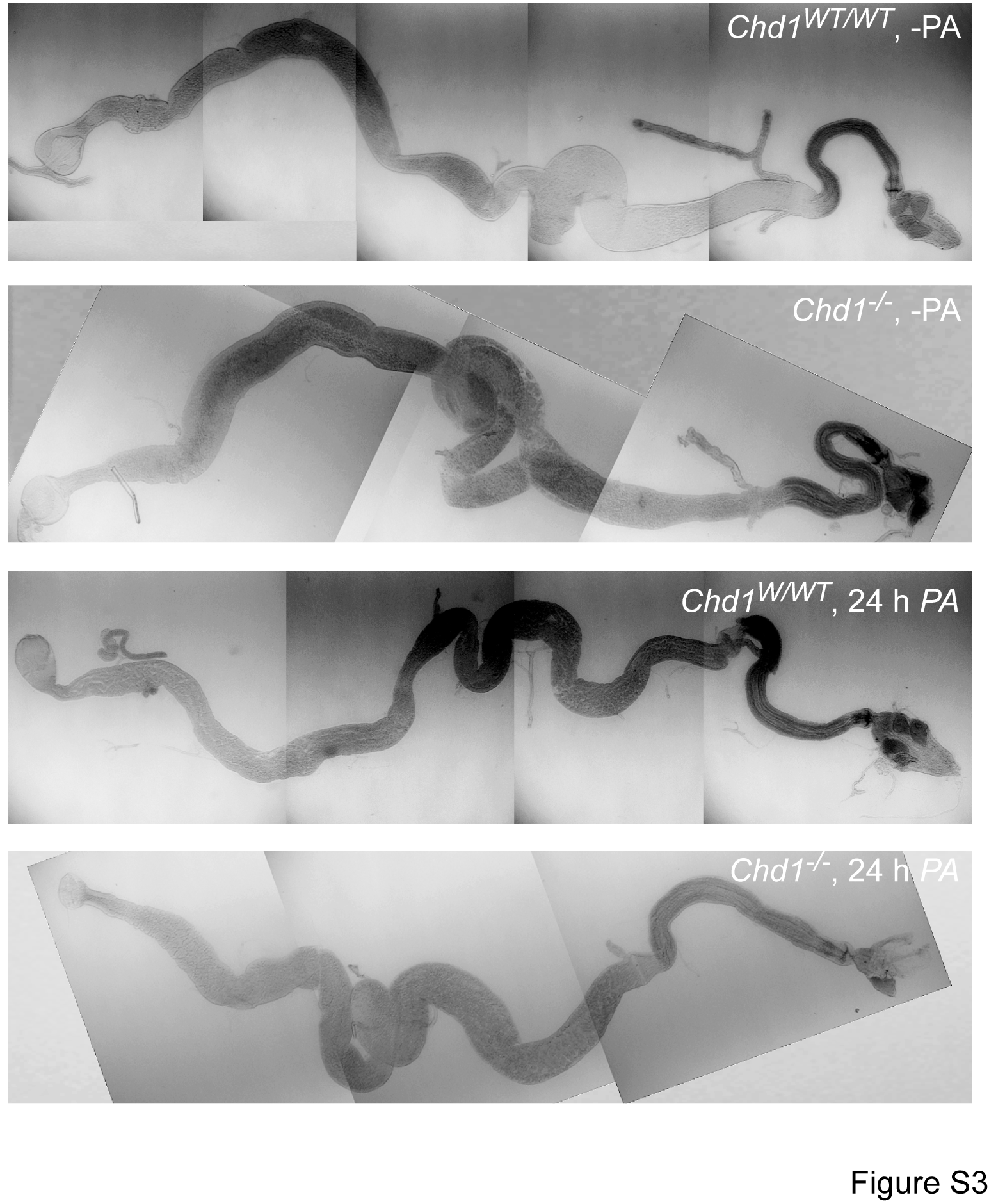

Supplement: Figure S3 — Overall gut morphology is similar in Chd1 -rescued and -mutant flies irrespective of the state of infection. Several light microscopic images were taken along the anterior posterior axis of dissected guts (midgut plus hindgut) and subsequently assembled into one picture using Photoshop. (TIF) [file pone.0043144.s003.tif]

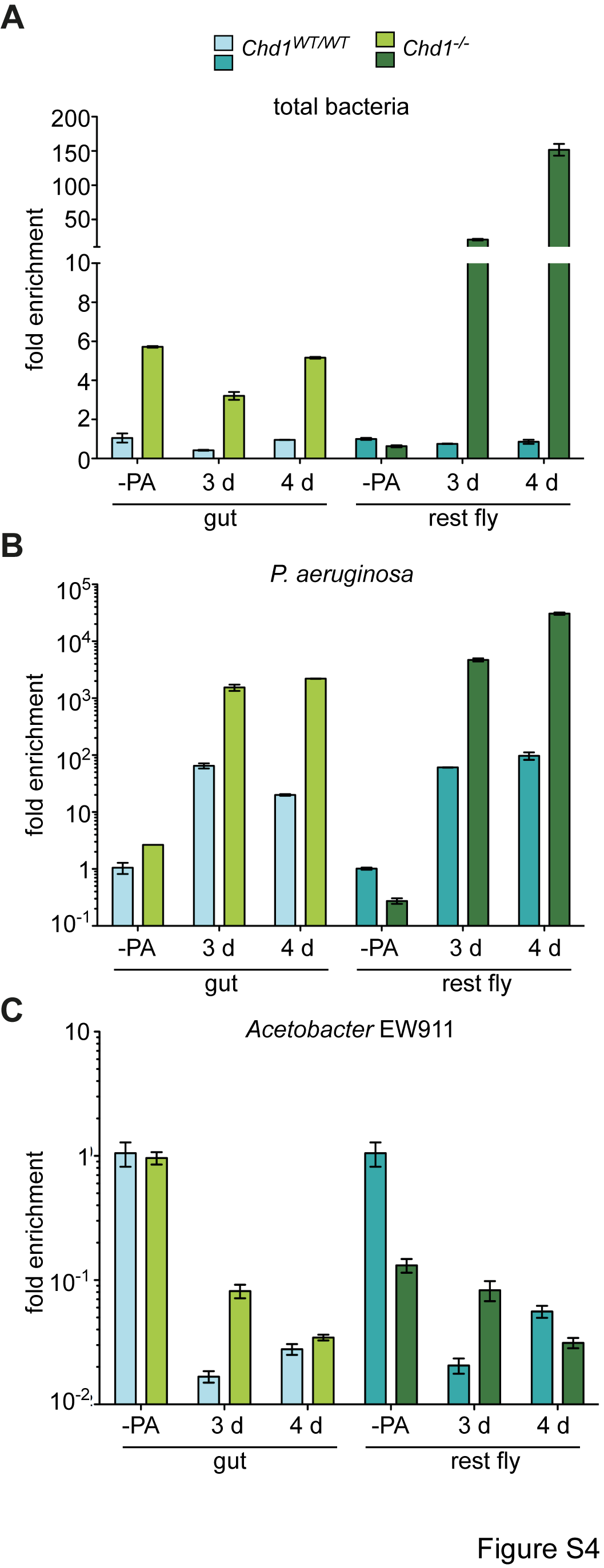

Supplement: Figure S4 — Bacterial load is elevated in Chd1 -mutant flies. (A) Bacterial load was analyzed in isolated guts and in whole flies from which intestines had been removed. qPCR was performed with primers targeting 16S rDNA in the absence of infection (−PA) as well as 3 days and 4 days after oral infection with P. aeruginosa. (B) 251658240P. aeruginosa titers are strongly increased in Chd1 −/− flies after infection. qPCR as in (A) with primers specific for P. aeruginosa. (C) Analysis of the gut-specific bacterium Acetobacter EW911. qPCR as in (A) with primers specific for Acetobacter EW911. The Drosophila Rpl32 gene was used for normalization, and enrichment relative to the non-infected (−PA) Chd1WT/WT line was calculated using the 2−ΔΔCT method. Values represent mean +/− SD of three independent experiments. (TIF) [file pone.0043144.s004.tif]

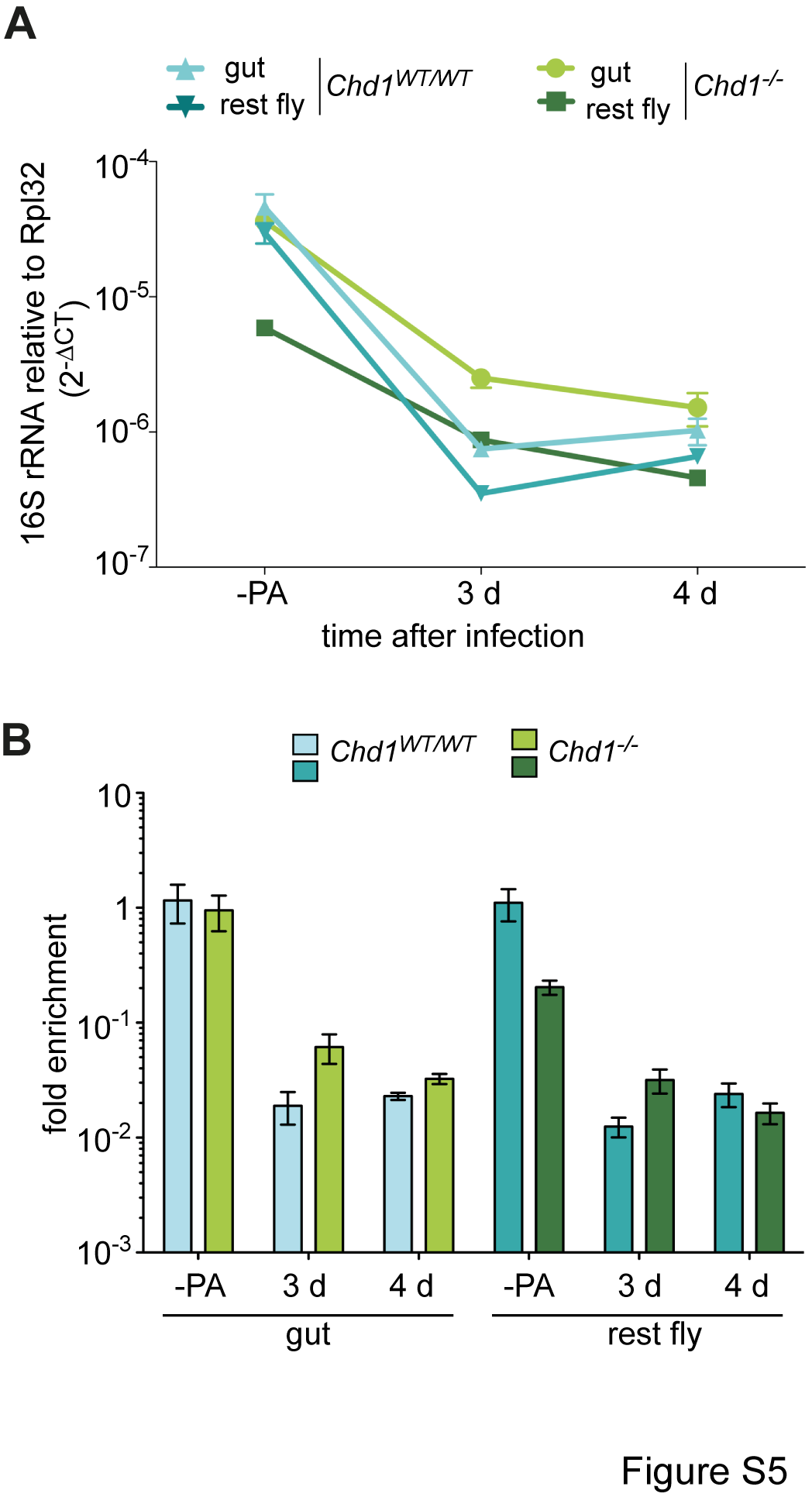

Supplement: Figure S5 — Analysis of the gut-specific bacterium Gluconobacter EW707. qPCR as in Figure S3 with primers specific for Gluconobacter EW707. (A) The relative differences of GB EW707 and the fly Rpl32 gene are expressed as 2−ΔCT values. (B) Enrichment relative to the non-infected (−PA) Chd1WT/WT line was calculated using the 2−ΔΔCT method. Values represent mean +/− SD of three independent experiments. (TIF) [file pone.0043144.s005.tif]

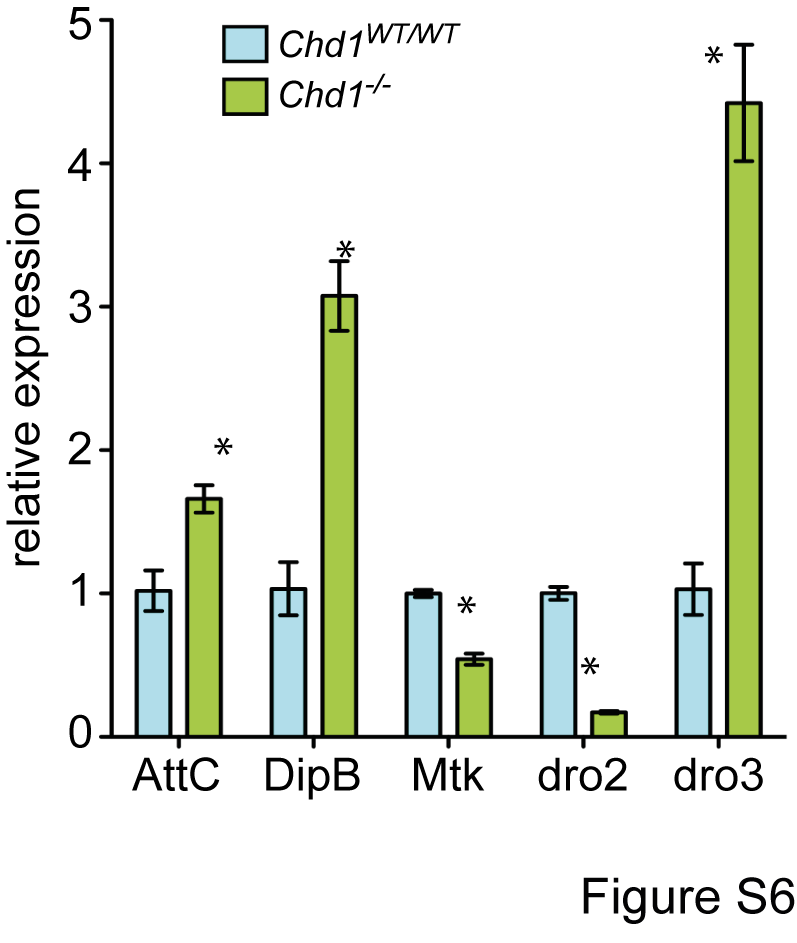

Supplement: Figure S6 — Several AMP genes are upregulated in Chd1 − /− larvae in the absence of infection. Indicated AMP genes that did not score in the microarray were analysed by RT-qPCR. Transcript levels were normalized against Rpl32 and are expressed relative to values obtained in Chd1WT/WT larvae. Values represent mean +/− SD of three independent experiments. Statistical significance was determined using unpaired t-test analysis (*P<0.05). (TIF) [file pone.0043144.s006.tif]
