# Supplementary material for: CHD1 Contributes to Intestinal Resistance against Infection by P. aeruginosa in Drosophila melanogaster
Source: PLoS One. 2012 Aug 13;7(8):e43144. doi: 10.1371/journal.pone.0043144 (PMC3418260; doi:10.1371/journal.pone.0043144)
Supplement: Table S1 — Upregulated genes in Chd1−/− flies. (PDF) [file pone.0043144.s007.pdf]

| Flybase ID | name | function | M-value | p-value |
|---|---|---|---|---|
| *detoxification* | | | | |
| CG2259-RA | Gclc | glutathione metabolic process | 1.03 | 0.00087498 |
| CG1681-RA | | glutathione transferase activity | 1.08 | 0.00064379 |
| CG15077-RA | Cyp12b2 | monooxygenase activity | 1.15 | 0.00441448 |
| CG14031-RA | Cyp4ac3 | oxidation-reduction process | 1.17 | 0.00043818 |
| CG6816-RB | Cyp18a1 | oxidation-reduction process | 1.19 | 0.00188902 |
| CG8864-RA | Cyp28a5 | oxidation-reduction process | 1.20 | 0.00055361 |
| CG13279-RA | Cyt-b5-r | oxidation-reduction process | 1.36 | 0.00526640 |
| CG4423-RA | Glutathione S transferase D6 | glutathione transferase activity | 1.42 | 0.00553136 |
| CG4623-RA | GDAP1 | Gst-like | 1.66 | 0.00064726 |
| CG3616-RA | Cyp9c1 | oxidation reduction | 1.77 | 1.41E-06 |
| CG13977-RA | Cyp6A18 | Cytochrome P450 | 1.93 | 0.03064446 |
| CG15902-RA | Ugt86Dj | UDP-glucuronosyl/UDP-glucosyltransferase; detoxification | 1.97 | 0.0022462 |
| CG17577-RA | Cyp9h1 | oxidation-reduction process | 2.02 | 0.00854499 |
| CG17530-RA | Glutathione S transferase E6 | detoxification | 2.06 | 0.00027271 |
| CG10093-RA | Cyp313a3 | oxidation-reduction process | 2.33 | 0.00041139 |
| CG10246-RA | Cyp6a9 | insecticide metabolic process | 2.40 | 1.80E-06 |
| CG4321-RA | Cyp4d8 | oxidation-reduction process; gut-specific expression | 2.52 | 0.00082261 |
| CG10242-RA | Cyp6a23 | oxidation-reduction process; gut-specific expression | 2.58 | 9.83E-05 |
| CG17531-RA | Glutathione S transferase E7 | glutathione transferase activity | 3.16 | 3.15E-05 |
| CG17527-RA | Glutathione S transferase E5 | glutathione transferase activity | 3.62 | 0.00013464 |
| CG9438-RA | Cyp6a2 | oxidation-reduction process | 4.93 | 9.09E-05 |
| CG1944-RA | Cyp4p2 | oxidation reduction; development related? | 5.33 | 2.09E-06 |
| CG4105-RA | Cyp4e3 | oxidation-reduction process | 6.19 | 0.00024667 |
| *development* | | | | |
| CG4715-RA | Iris | oogenesis | 1.00 | 0.08008523 |
| CG4559-RA | Imaginal disc growth factor 3 | imaginal disc development | 1.00 | 0.00094915 |
| CG9325-RD | Hu li tai shao | female germline development | 1.03 | 0.00266471 |
| CG13461-RA | | ecdysone target? | 1.10 | 0.15598479 |
| CG3151-RA | RNA-binding protein 9 | establishment of blood-brain barrier | 1.16 | 8.55E-05 |
| CG6339-RA | Rad50 | instar larval development | 1.18 | 1.15E-06 |
| CG1546-RA | Prolyl-4-hydroxylase-alpha SG2 | salivary gland morphogenesis | 1.23 | 0.00058748 |
| CG33197-RB | Muscleblind | compound eye photoreceptor cell differentiation | 1.29 | 0.07196156 |
| CR_TC_RE65113 | Blistery | wing morphogenesis | 1.33 | 0.00087371 |
| CG5740-RA | | sexual differentiation? | 1.33 | 0.00030361 |
| CG15784-RA | | germ cell development? | 1.42 | 0.00047889 |
| CG12092-RA | NPC1b | nervous system development | 1.47 | 0.01562287 |
| CG4760-RC | Boule | mRNA binding; spermatogenesis | 1.54 | 0.00147809 |
| CG9480-RB | Glycogenin | mesoderm development | 1.56 | 0.00841134 |
| CG12052-RF | Lola | txn factor; neural development? | 1.64 | 0.00633794 |
| CG14265-RB | | ecdysone response? | 1.75 | 0.00058178 |
| CG31666-RD | Chinmo | txn factor; effector of JAK/STAT pathway | 1.79 | 0.00028876 |
| CG15444-RB | Inebriated | neural development | 1.81 | 0.00942179 |
| CG32120-RA | Senseless | organ morphogenesis; organ development | 1.84 | 7.55E-08 |

| Flybase ID | name | function | M-value | p-value |
|---|---|---|---|---|
| CG11405-RA | Activating txn factor 3 | neuronal development, epithelial replacement | 1.93 | 0.00051653 |
| CG3767-RA | JhI-26 | developmentally regulated? | 1.94 | 0.00093578 |
| CG30021-RA | Skiff | guanylate kinase domain; germ line development? | 1.95 | 2.12E-05 |
| CG5867-RA | | juvenile hormone binding motif | 1.99 | 0.000553 |
| CG7529-RA | Esterase Q | involved in juvenile hormone degradation? | 2.02 | 0.00027737 |
| LD11885 | Ftz-f1 | cDNA clone of Ftz-f1 transcription factor; metamorphosis | 2.02 | 0.00021628 |
| CG12370-RA | Diuretic hormone 44 receptor 1 | neuropeptide receptor | 2.08 | 0.00113617 |
| CG31666-RC | Chinmo | transcription factor; JAK/STAT pathway; stem cell renewal | 2.12 | 0.00108177 |
| CG10918-RA | | ecdysone target | 2.25 | 2.29E-05 |
| CG7390-RB | Senescence marker protein-30 | germ line development; circadian behaviour | 2.25 | 0.0003463 |
| CG5441-RA | Delilah | mesoderm & muscle cell development | 2.28 | 0.0013199 |
| CG14307-RC | Fruitless | multicellular organismal development | 2.36 | 0.0012122 |
| CG7390-RA | Senescence marker protein-30 | germ line development; circadian behaviour | 2.45 | 5.23E-05 |
| CG32694-RA | | developmentally regulated? | 2.47 | 0.00040137 |
| CG18251-RB | Muscle-specific protein 300 | skeletal muscle tissue development | 2.52 | 0.00010398 |
| CG12794-RA | La costa | development?; starvation?; cell division | 2.78 | 1.72E-05 |
| LP09368 | | glue protein related | 2.99 | 0.00129374 |
| CG32071-RA | | male germline development? | 3.03 | 0.00260146 |
| CG9511-RA | | development? | 5.40 | 4.57E-05 |
| CG16898-RA | | protein kinase of unknown function; mating? | 6.11 | 5.77E-06 |
| CG4533-RB | Lethal (2) essential for life | response to heat (Hsp20-like); embryonic development | 6.42 | 5.13E-08 |
| *immune response* | | | | |
| CG12199-RC | Kekkon5 | immune response, Hox-target?; regulation of BMP signaling pathway | 1.01 | 0.0390069 |
| CG7905-RA | Ect4 | signal transduction; innate immune response | 1.04 | 0.00989182 |
| CG16705-RA | SPE | Toll signaling pathway | 1.13 | 0.0053839 |
| CG12844-RA | Tetraspanin 42Eh | immune defense; activation of NF-KB & JNK | 1.14 | 0.0009265 |
| CG4099-RA | Sr-CI | scavenger receptor activity; immune response | 1.30 | 0.00362589 |
| CG8562-RA | | carboxypeptidase, immune response | 1.36 | 0.00960536 |
| CG6667-RC | Dorsal | defense against gram-positive bacteria (Toll-pathway) | 1.36 | 0.00105667 |
| CG4920-RA | Easter | serine-type endopeptidase activity; Toll signaling pathway | 1.38 | 0.00972536 |
| CG12142-RA | Tetraspanin 42Eg | immune defense; activation of NF-KB & JNK | 1.39 | 0.00025045 |
| CG31783-RA | NinaD | scavenger receptor activity; defense response | 1.44 | 0.01692856 |
| CG11390-RA | Ejaculatory bulb protein III | response to virus; metamorphosis | 1.51 | 0.00025237 |
| CG16997-RA | Sp147 | serine endopeptidase; chymotrypsin-like; infection with sigma virus | 1.51 | 0.00010282 |
| CG2505-RA | Alpha Est2 | carboxylesterase; immune response? | 1.51 | 2.81E-07 |
| CG11242-RA | | cytoskeleton associated; NFkappaB target? | 1.57 | 1.82E-05 |
| CG10433-RA | | involved in defense | 1.61 | 0.00010137 |
| CG5597-RA | | immunglobuline like | 1.62 | 8.13E-06 |
| CG7915-RB | Ect-4 | membrane receptor; signalling; innate immune response | 1.64 | 0.00062502 |
| CG33119-RA | Nimrod B1 | phagocytosis receptor with EGF repeats | 1.67 | 0.00042387 |
| CG15255-RA | | metalloprotease; gutspecific expression; oral immune response | 1.79 | 0.00814339 |
| CG8942-RA | NimrodC1 | wnt binding, wnt signalling; phagocytosis receptor | 1.81 | 0.00014419 |
| CG3775-RA | | metalloprotease; immune-specific regulation; development | 1.82 | 1.02E-06 |
| CG5791-RA | | innate immune response (kappaB sequence); Toll/Imd pathway | 1.84 | 0.01796389 |

| Flybase ID | name | function | M-value | p-value |
|---|---|---|---|---|
| CG2071-RA | Ser6 | serine endopeptidase; response to infection with sigma virus | 1.92 | 0.01520132 |
| CG13947-RA | | transcriptional response to infection with the sigma virus; mating | 2.05 | 0.00020219 |
| CG7248-RA | | chitin binding, peritrophic matrix; gut immune response | 2.08 | 0.00011169 |
| CG5550-RA | Fibrinogen-related protein | intestinal response to bacterial infection | 2.13 | 0.03147197 |
| CG8577-RA | PGRP-SC1b | peptidoglycan binding protein; Imd pathway | 2.16 | 0.00629333 |
| CG15293-RA | | hemolymph clotting factor like? (infection with the sigma virus) | 2.17 | 4.46E-06 |
| CG8620-RA | | intestinal response to bacterial infection | 2.29 | 0.00071804 |
| CG32475-RA | Methuselah-like 8 | response to stress, G-protein coupled receptor protein signaling | 2.32 | 7.36E-05 |
| CG17974-RA | | extracellular; involved in male fertility, immunity | 2.45 | 0.00075726 |
| CG9675-RA | Spheroide | serine peptidase; immune response; stress response | 2.52 | 0.00035713 |
| CG13324-RA | | immune response ? | 2.53 | 0.00315386 |
| CG9928-RA | | immune response; cell cycle progression; circadian behaviour? | 2.55 | 9.10E-05 |
| CG31508-RA | Turandot C | humoral factor; induced by stress and infection | 2.62 | 0.00809598 |
| CG18179-RA | | serine peptidase; immune response; stress response | 2.66 | 0.00093054 |
| CG18331 | Mucin 68Ca | blood coagulation; glue protein related; expressed in gut | 2.88 | 0.00146065 |
| CG33178-RA | | glutathion metabolism? | 2.88 | 0.00010361 |
| CG5778-RA | | innate immune response (kappaB target); Toll/Imd pathway | 2.92 | 0.0014496 |
| CG3174-RA | Fmo-2 | oxidation reduction; response to parasitoid attack | 3.17 | 0.00096201 |
| CG14419-RA | | host defense? | 3.60 | 0.00156509 |
| CG16775-RA | | host defense to Pseudomonas infection | 3.80 | 0.00090638 |
| CG11765-RA | Peroxiredoxin 2540 | oxidative stress; gut-specific immunity? | 4.76 | 3.61E-05 |
| CG8523-RA | Multi drug resistance 50 | drug transport (ABC-transporter); gut-specific expression | 5.77 | 4.65E-08 |

*intracellular processes*

| Flybase ID | name | function | M-value | p-value |
|---|---|---|---|---|
| CG14066-RB | La related protein | autophagic cell death; spindle assembly in male meiosis | 1.01 | 0.0018697 |
| CG8266-RA | Sec31 | RNA splicing | 1.01 | 3.39E-05 |
| CG10798-RA | Diminutive | regulation of transcription | 1.02 | 0.00445911 |
| CG5753-RA | Staufen | mRNA localization | 1.04 | 0.00182449 |
| CG18124-RA | MTTF | transcription termination factor activity | 1.07 | 0.0003383 |
| CG31005-RA | Qless | cellular response to stress; negative regulation of apoptosis | 1.07 | 0.00038706 |
| CG4675-RA | Na[+]-driven anion exchanger 1 | inorganic anion exchanger activity | 1.07 | 0.00237041 |
| CG8318-RA | Neurofibromin 1 | response to stress | 1.10 | 0.00359294 |
| CG8503-RA | | negative regulation of transcription | 1.16 | 0.00136588 |
| CG32384-RA | Formin 3 | actin binding | 1.19 | 0.00190956 |
| CG31649-RA | Muscle-specific protein 300 | actin binding | 1.25 | 0.00187558 |
| CG5599-RA | | phagocytosis; engulfment | 1.27 | 0.00215329 |
| CG33197-RA | Muscleblind | apoptosis | 1.34 | 0.03838336 |
| CG2681-RA | | protein ubiquitination | 1.42 | 0.01210384 |
| CG5247-RA | Inverted repeat-binding protein | ATP-dependent DNA helicase activity | 1.44 | 0.00165099 |
| CG3328-RA | | regulation of transcription | 1.46 | 0.00030354 |
| CG4226-RB | Glutamate receptor IIC | synaptic transmission; establishment of protein localization | 1.46 | 0.00024776 |
| CG11205-RA | Phr | DNA repair | 1.48 | 0.33756872 |
| CG11797-RA | Odorant-binding protein 56a | sensory perception of smell | 1.48 | 3.11E-05 |
| CG5205-RB | | DNA/RNA helicase (DEAD/DEAH box type); mRNA metabolism | 1.53 | 0.00716211 |
| CG6043-RB | | HMG box, mating responsive genes | 1.54 | 0.00052955 |

| Flybase ID | name | function | M-value | p-value |
|---|---|---|---|---|
| HDC11981 | Diver | transposon | 1.60 | 1.66E-05 |
| CG30118-RA | Pms2 | mismatch repair; ATP binding | 1.60 | 4.36E-06 |
| CG6503-RA | | putative non-coding RNA | 1.61 | 0.00038805 |
| CG10788-RB | New glue 3 | puparial adhesion; ecdysone regulated | 1.61 | 0.0003735 |
| CG32307-RA | Zormin | muscle cytosceleton; chromosome movement | 1.74 | 0.0046061 |
| CG8144-RB | Pasilla | mRNA splicing | 1.74 | 0.00440885 |
| CG32922-RB | Skeletor | spindle assembly | 1.80 | 0.00029041 |
| CG14661-RA | | hormone binding? | 1.88 | 0.00091458 |
| CG6910-RA | | stress response? | 1.90 | 0.0001109 |
| CG12863-RA | | zinc finger containing; DNA methylase domain | 1.92 | 0.00030675 |
| CG30285-RA | | RNA binding? | 2.12 | 1.74E-05 |
| CG14872-RB | | lipocalin like; starvation response? | 2.29 | 0.00209701 |
| CG18410-RA | | RuV A like | 2.33 | 9.28E-05 |
| CG2120-RA | | zinc finger; nuclear function? | 2.40 | 0.00035613 |
| CG14872-RA | | calycin like; starvation, stress? | 2.45 | 0.00407466 |
| CG11909-RA | Target of brain insulin | alpha-Glucosidase; stress response? | 2.57 | 0.00063953 |
| CG6839-RA | | endonuclease | 2.60 | 3.40E-05 |
| CG7299-RA | | circadian behavior? | 2.73 | 0.00044361 |
| CG11659-RA | | actin binding; starvation linked? | 2.85 | 0.02962492 |
| CG5070-RA | | PcG target? | 3.12 | 0.00163486 |
| CG9757-RA | | starvation linked? | 4.07 | 0.00031618 |
| *metabolism* | | | | |
| CG30427-RB | | oxidation-reduction process | 1.00 | 0.02638294 |
| CG7149-RA | | phospholipid biosynthetic process; phagocytosis | 1.00 | 0.00970115 |
| CG11727-RB | | regulation of Rab GTPase activity | 1.01 | 0.00955055 |
| CG3902-RA | | oxidation-reduction process | 1.02 | 0.00045104 |
| CG11453-RA | | metabolic process | 1.03 | 2.94E-05 |
| CG15534-RA | | sphingomyelin metabolic process | 1.08 | 0.11069712 |
| CG3869-RA | Marf | GTPase activity | 1.08 | 0.00060859 |
| CG40486-RB | | oxidation-reduction process | 1.09 | 1.37E-05 |
| CG31332-RC | Unc-115 | ion binding | 1.10 | 0.00010277 |
| CG9674-RD | | oxidation-reduction process | 1.11 | 0.00022751 |
| CG3523-RA | | fatty acid synthase activity | 1.11 | 0.0028913 |
| CG6733-RA | | aminoacylase activity | 1.11 | 0.00106086 |
| CG2194-RB | Rhythmically expressed gene 3 | oxidation-reduction process | 1.12 | 0.00586182 |
| CG10120-RB | Malic enzyme | malate metabolic process | 1.14 | 0.00259536 |
| CG18730-RA | Amy-p | alpha-amylase activity | 1.14 | 0.00267996 |
| CG1131-RA | Alpha-Esterase-10 | carboxylesterase activity | 1.15 | 0.00408333 |
| CG30492-RC | | zinc ion binding | 1.15 | 0.00048888 |
| CG32645-RB | | transferase activity | 1.15 | 1.17E-05 |
| CG31956-RA | Pgant4 | polypeptide N-acetylgalactosaminyltransferase activity | 1.15 | 3.92E-06 |
| CG17322-RD | | UDP-glycosyltransferase | 1.15 | 0.05364296 |
| CG31475-RA | | calcium ion binding | 1.16 | 0.03497045 |
| CG17273-RA | | adenylosuccinate synthase activity | 1.16 | 0.00014543 |

| Flybase ID | name | function | M-value | p-value |
|---|---|---|---|---|
| CG9363-RB | | tyrosine catabolic process; L-phenylalanine catabolic process | 1.17 | 0.00383555 |
| CG8772-RD | No extended memory | carbon-monoxide oxygenase activity | 1.18 | 0.00110832 |
| CG18578-RA | Ugt86Da | glucuronosyltransferase activity | 1.19 | 0.00040566 |
| CG11967-RA | CAHbeta | carbonate dehydratase activity | 1.20 | 0.00035498 |
| CG7920-RA | | acetyl-CoA metabolic process | 1.20 | 0.0004567 |
| CG17725-RB | Pepck | phosphoenolpyruvate carboxykinase (GTP) activity | 1.20 | 0.00040589 |
| CG3926-RA | Spat | serine-pyruvate transaminase activity | 1.21 | 0.00012923 |
| CG33048-RA | Mocs1 | metal ion binding | 1.21 | 0.00151766 |
| CG32649-RA | | protein kinase activity | 1.21 | 0.00287717 |
| CG6622-RB | Protein C kinase 53E | protein serine/threonine kinase activity | 1.22 | 0.00808031 |
| CG14655-RA | | zinc ion binding | 1.22 | 4.55E-05 |
| CG13397-RA | | alpha-N-acetylglucosaminidase activity | 1.22 | 0.00027961 |
| CG8129-RB | | L-threonine ammonia-lyase activity | 1.23 | 0.00045666 |
| CG10564-RA | Adenylyl cyclase 78C | adenylate cyclase activity | 1.25 | 0.0007867 |
| CG10723-RA | Kua | ubiquitination | 1.25 | 0.00045121 |
| CG3725-RA | Calcium ATPase at 60A | calcium-transporting ATPase activity | 1.28 | 0.01674941 |
| CG11162-RA | | oxidation-reduction process | 1.28 | 0.01482666 |
| CG32557-RA | | oxidation-reduction process | 1.30 | 0.01140695 |
| CG7171-RA | Urate oxidase | urate oxidase activity | 1.31 | 0.00249443 |
| CG3597-RA | | oxidation-reduction process | 1.31 | 0.00256665 |
| CG3301-RA | | oxidation-reduction process | 1.31 | 9.44E-05 |
| CG3752-RA | Aldehyde dehydrogenase | aldehyde dehydrogenase (NAD) activity | 1.31 | 0.00031011 |
| CG5704-RA | | catalytic activity | 1.32 | 0.00685119 |
| CG1315-RA | | arginine biosynthetic process | 1.33 | 0.00294828 |
| CG6644-RA | UDP-glycosyltransferase 35a | UDP-glycosyltransferase activity | 1.34 | 0.00066048 |
| CG5652-RA | | digestive system process | 1.35 | 0.0176111 |
| CG11198-RA | ACC | fatty acid biosynthetic process | 1.36 | 0.00105605 |
| CG30445-RA | Tyrosine decarboxylase 1 | tyrosine decarboxylase activity | 1.36 | 0.00436727 |
| CG1673-RA | | branched chain family amino acid biosynthetic process | 1.37 | 0.00115857 |
| CG18522-RA | | oxidoreductase activity | 1.38 | 0.00011817 |
| CG9042-RB | Gpdh | glycerol-3-phosphate dehydrogenase [NAD+] activity | 1.39 | 0.00296573 |
| CG1774-RA | | cellular metabolic process | 1.40 | 0.00237636 |
| CG10175-RA | | carboxylesterase activity | 1.41 | 0.00355328 |
| CG15106-RA | Jheh3 | epoxide hydrolase activity | 1.42 | 1.42E-05 |
| CG10827-RA | | alkaline phosphatase activity | 1.42 | 0.00053835 |
| CG14994-RB | Glutamic acid decarboxylase 1 | glutamate decarboxylase activity | 1.45 | 6.67E-05 |
| CG6917-RA | Esterase 6 | pheromone biosynthetic process; courtship behavior | 1.47 | 0.02792752 |
| CG33093-RA | | oxidation-reduction process | 1.48 | 0.0725207 |
| CG6074-RA | | carbonate dehydratase activity | 1.48 | 0.00010434 |
| CG7525-RA | Tie-like receptor tyrosine kinase | protein tyrosine kinase activity | 1.49 | 0.00114062 |
| CG8128-RA | CG8128 | hydrolase activity | 1.50 | 0.00924027 |
| CG1121-RA | Alpha-Esterase-8 | carboxylesterase activity | 1.50 | 0.01089965 |
| CG5707-RA | | hydrolase activity | 1.52 | 0.00316561 |
| CG13311-RA | | alkaline phosphatase-like | 1.53 | 0.00228151 |
| CG8462-RA | Obp56e | odorant binding protein | 1.53 | 0.00026202 |

| Flybase ID | name | function | M-value | p-value |
|---|---|---|---|---|
| CG14616-RE | | histidine phosphatase | 1.53 | 0.00231184 |
| CG14787-RA | | crotonase | 1.54 | 0.00022308 |
| CG3841-RA | | carboxylesterase | 1.56 | 0.00033739 |
| CG9993-RA | | AMP-dependent synthetase/ligase | 1.57 | 0.00511214 |
| CG1915-RA | Sallimus | large protein; muscle physiology | 1.63 | 0.00462844 |
| CG30503-RA | | lipid catabolic process; phospholipase A motif | 1.65 | 0.00106932 |
| CG14762-RA | | protein binding (leu rich repeat) | 1.66 | 0.00198618 |
| CG10165-RA | | cyt b561 type; ferric reductase | 1.66 | 0.00044022 |
| CG9619-RA | | regulatory phosphatase? | 1.67 | 0.00057154 |
| CG4772-RA | Ugt86Dh | glucuronosyltransferase activity | 1.72 | 0.00035339 |
| CG7025-RA | | carboxypeptidase | 1.72 | 0.00098037 |
| CG6816-RA | Cyp18a1 | steroid biosynthetic process | 1.73 | 0.0044973 |
| CG11315-RB | Niemann-Pick type C-2h | sterol homeostasis and steroid biosynthesis | 1.73 | 0.00152066 |
| CG8256-RC | Gpo-1 | glycerophosphate oxidase | 1.73 | 0.00299337 |
| CG31719-RA | Rlua-1 | pseudouridine synthase | 1.74 | 6.69E-05 |
| CG1615-RB | Ork1 | K+ channel; neural physiology | 1.75 | 0.00196548 |
| CG4123-RB | Mipp 1 | multiple inositol polyphosphate phosphatase | 1.75 | 0.00179174 |
| CG15879-RA | | lipase motif | 1.76 | 0.0004654 |
| CG5156-RA | | acyltransferase | 1.77 | 0.0004503 |
| CG9466-RA | | mannose metabolic process | 1.80 | 0.0194169 |
| CG8708-RB | | protein glycosylation | 1.84 | 0.00169237 |
| CG3835-RB | | FAD-linked oxidase domain | 1.84 | 6.39E-06 |
| CG1915-RC | Sallimus | large protein; muscle physiology | 1.86 | 0.00407212 |
| CG30502-RA | | cytochrome b5; fatty acid biosynthetic process | 1.87 | 0.0049469 |
| CG1461-RA | | aromatic amino acid family metabolic process | 1.87 | 0.00146335 |
| CG8629-RA | | acyl CoA binding | 1.96 | 0.00144941 |
| CG10592-RA | | alkaline phosphatase-like; digestion? | 1.99 | 0.00048385 |
| CG15117-RA | | beta glucuronidase, carbohydrate metabolism | 2.02 | 8.25E-05 |
| CG32170-RA | | oxidoreductase | 2.02 | 0.0006751 |
| CG30171-RB | Unc-89 | myosin light chain kinase; muscle physiology | 2.02 | 0.0154246 |
| CG5431-RA | | sulfotransferase domain; tissue regeneration? | 2.03 | 0.00280518 |
| CG9042-RA | Gpdh | triglyceride metabolic process; flight behavior | 2.10 | 0.00052917 |
| CG2184-RA | Myosin light chain 2 | ATPase activity | 2.10 | 0.00137081 |
| CG18104-RA | Arginase | arginase | 2.12 | 0.00020222 |
| CG7464-RA | Chitin synthase 2 | chitin synthase activity | 2.17 | 0.0009636 |
| CG17843-RA | | cell redox homeostasis, thioredoxin-like; seminal fluid | 2.17 | 3.31E-05 |
| CG18519-RA | | FE-S-cluster, iron binding | 2.23 | 0.00140255 |
| CG9734-RC | | hemoglobin, oxygen transport | 2.24 | 9.64E-05 |
| CG30457-RA | | storage protein? | 2.26 | 0.02020778 |
| CG9743-RA | | lipid metabolic process; oxidation-reduction process | 2.35 | 0.00036899 |
| CG32751-RA | | nitrogen compound metabolic process | 2.36 | 0.00076416 |
| CG9468-RA | | mannose metabolic process; glycan degradation | 2.36 | 0.01029935 |
| CG4753-RB | | phospholipid/glycerol acyltransferase; immune response? | 2.36 | 0.00099308 |
| CG11126-RA | | nucleotide catabolic process; PcG-target? | 2.40 | 0.00206482 |
| CG12512-RA | | long-chain-fatty-acid-CoA ligase; immune response? | 2.41 | 0.00012077 |

| Flybase ID | name | function | M-value | p-value |
|---|---|---|---|---|
| CG15828-RA | | lipid binding; microtubule association | 2.51 | 0.00042443 |
| CG5150-RA | | alkaline phosphatase-like; digestion?; gutspecific expression | 2.65 | 0.00048158 |
| CG32754-RA | Vanin-like | nitrogen compound metabolic process | 2.92 | 0.00112392 |
| CG8630-RA | | lipid metabolic process; oxidation-reduction; immune response | 3.04 | 0.00043691 |
| CG3106-RA | | acetyltransferase family; starvation linked? | 3.11 | 0.00179563 |
| CG14022-RA | | acyl phosphatase | 3.12 | 1.93E-05 |
| CG8719-RA | Ornithine decarboxylase 2 | polyamine biosynthetic process | 3.24 | 3.02E-06 |
| CG14205-RA | | transferase; starvation linked? | 3.74 | 0.00060033 |
| CG7526-RA | | calcium ion binding; transmembran receptor | 4.34 | 0.00039006 |
| CG8424-RA | Jhedup | carboxylesterase activity | 4.41 | 5.59E-05 |
| CG11796-RA | | aromatic amino acid family metabolic process | 4.52 | 0.00016133 |
| CG4779-RA | Hgo | L-phenylalanine catabolic process; tyrosine catabolic process | 4.55 | 4.47E-06 |
| CG4716-RA | | methylenetetrahydrofolate dehydrogenase activity | 5.00 | 1.72E-08 |
| CG4716-RB | | methylene tetrahydrofolate reductase | 5.95 | 1.19E-07 |
| CG4757-RA | | carboxylesterase activity | 7.57 | 2.27E-05 |
| *proteolysis* | | | | |
| CG9505-RA | | metalloendopeptidase activity | 1.00 | 0.00244091 |
| CG18417-RA | | metallocarboxypeptidase activity | 1.01 | 0.06139854 |
| CG31821-RA | | proteolysis | 1.06 | 9.72E-05 |
| CG9564-RA | Trypsin 29F | serine-type endopeptidase activity | 1.07 | 0.02656151 |
| CG18493-RA | | serine-type peptidase activity | 1.10 | 0.01695659 |
| CG4721-RA | | metalloendopeptidase activity | 1.10 | 0.00012724 |
| CG17739-RA | | serine-type endopeptidase inhibitor activity | 1.10 | 0.07439514 |
| CG4725-RA | | metalloendopeptidase activity | 1.14 | 0.00025689 |
| CG8342-RA | E(spl) region transcript m1 | serine-type endopeptidase inhibitor activity | 1.20 | 8.48E-05 |
| CG7532-RA | L(2)34Fc | serine-type endopeptidase activity | 1.22 | 0.00076501 |
| CG11066-RB | Scarface | serine-type endopeptidase activity | 1.22 | 0.00311658 |
| CG6225-RA | | aminopeptidase activity | 1.25 | 0.00763476 |
| CG16712-RA | | serine-type endopeptidase inhibitor activity | 1.31 | 0.00013375 |
| CG13095-RA | Bace | aspartic-type endopeptidase activity | 1.31 | 0.00138281 |
| CG7754-RA | IotaTrypsin | serine-type endopeptidase activity | 1.32 | 1.32E-05 |
| CG32130-RA | Starvin | serine peptidase | 1.34 | 0.00229902 |
| CG17477-RA | | serine-type endopeptidase activity | 1.37 | 0.01254035 |
| CG30043-RA | | proteolysis | 1.41 | 0.00048459 |
| CG6337-RA | | cysteine-type endopeptidase activity | 1.44 | 1.03E-05 |
| CG32483-RA | | serine-type carboxypeptidase activity | 1.47 | 0.00379016 |
| CG6048-RA | Sp108 | serine endopeptidase; chymotrypsin-like | 1.52 | 2.84E-06 |
| CG5246-RA | | serine endopeptidase | 1.54 | 0.00082942 |
| CG3604-RA | | serine endopeptidase | 1.54 | 8.57E-05 |
| CG12387-RA | Zeta Trypsin | serine endopeptidase; chymotrypsin-like; gutspecific expression | 1.58 | 1.20E-05 |
| CG5905-RB | Nep1 | metalloprotease | 1.60 | 0.00014391 |
| CG4053-RA | | serine endopeptidase; chymotrypsin-like | 1.63 | 6.69E-05 |
| CG10472-RA | | serine endopeptidase | 1.66 | 8.71E-05 |
| CG11961-RB | | metalloprotease; predominantly expressed in gut | 1.70 | 0.00039023 |

| Flybase ID | name | function | M-value | p-value |
|---|---|---|---|---|
| CG17475-RA | | serine endopeptidase; chymotrypsin-like; gutspecific expression | 1.70 | 0.00119576 |
| CG13160-RA | | metalloprotease | 1.77 | 0.00054436 |
| CG17134-RA | | aspartic type endopeptidase | 1.78 | 4.62E-05 |
| CG10051-RA | | metalloprotease; gutspecific expression | 1.93 | 0.00012911 |
| CG33127-RA | | serine endopeptidase; gutspecific expression | 1.94 | 0.00031433 |
| CG33459-RA | | serine endopeptidase | 1.95 | 0.00016597 |
| CG30371-RA | | serine endopeptidase | 2.03 | 0.00102864 |
| CG31233-RA | | amino peptidase activity | 2.05 | 0.00173738 |
| CG12388-RA | | peptidase cysteine/serine, kappa trypsin; gutspecific expression | 2.13 | 0.00257053 |
| CG4653-RA | | serine endopeptidase | 2.14 | 2.78E-05 |
| CG31343-RA | | peptidase; stress response?; gutspecific expression | 2.14 | 0.0016206 |
| CG12350-RA | Lambda Trypsin | peptidase cysteine/serine, lambda trypsin | 2.16 | 0.02982919 |
| CG33329-RB | Sp212 | serine endopeptidase; chymotrypsin-like | 2.27 | 0.00099957 |
| CG1304-RA | | serine endopeptidase | 2.42 | 0.01007348 |
| CG3513-RA | | protease inhibitor | 2.42 | 0.00016805 |
| CG31198-RA | | amino peptidase activity | 2.48 | 6.26E-05 |
| CG8774-RB | | aminopeptidase; predominantly expressed in gut | 2.95 | 0.00150022 |
| CG7722-RA | | protease inhibitor; serpin; development? | 2.95 | 1.62E-06 |
| CG14820-RA | | peptidase, protease inhibitor; gutspecific expression | 3.00 | 0.00014305 |
| CG31265-RA | | serine endopeptidase | 3.18 | 9.72E-05 |
| CG15254-RA | | metalloendopeptidase; sperm development? | 3.88 | 0.00035006 |

*signaling*

| | | | | |
|---|---|---|---|---|
| CG7125-RA | Protein Kinase D | protein serine/threonine kinase activity;  intracellular signaling | 1.03 | 0.00013456 |
| CG4322-RA | Moody | G-protein coupled receptor protein signaling pathway | 1.04 | 0.00017939 |
| CG3985-RB | Synapsin | neurotransmitter secretion | 1.05 | 0.02096796 |
| CG3665-RB | Fasciclin 2 | negative regulation of epidermal growth factor receptor signaling | 1.08 | 0.01344763 |
| CG16992-RA | Methuselah-like 6 | G-protein coupled receptor protein signaling pathway | 1.10 | 0.00022868 |
| CG10698-RA | GRHRII | G-protein coupled receptor protein signaling pathway | 1.11 | 0.00125151 |
| CG9538-RA | Antigen 5-related | hormone response? | 1.13 | 0.00015327 |
| CG14734-RA | Tachykinin | neuropeptide signaling pathway | 1.15 | 0.00400984 |
| CG12232-RA | G protein alpha 73B | GTPase activity | 1.20 | 0.00111772 |
| CG8604-RA | Amphiphysin | neurotransmitter secretion | 1.23 | 0.00270433 |
| CG32843-RA | | G-protein coupled receptor protein signaling pathway | 1.32 | 0.00020899 |
| CG12290-RA | | G-protein coupled receptor protein signaling pathway | 1.36 | 0.03951639 |
| CG7054-RA | | phosphatidyl ethanolamin binding, signalling? | 1.41 | 0.00159467 |
| CG10524-RA | Protein kinase C delta | protein serine/threonine kinase activity | 1.44 | 0.00054744 |
| CG15529-RA | | SH2 domain; signalling pathway? | 1.50 | 5.74E-06 |
| CG33146-RA | Mctp | transmembrane protein; signalling | 1.56 | 0.00040352 |
| CG8279-RA | Phosphodiesterase 6 | signalling | 1.56 | 0.00022666 |
| CG5402-RA | | Wnt binding, Wnt signalling | 1.76 | 8.49E-05 |
| CG7978-RA | Adenyl cyclase 76E | intracellular signalling | 1.78 | 0.00297145 |
| CG8907-RA | | src homology domain; ras signaling? | 1.80 | 0.0006971 |
| CG9498-RA | | CHK kinase-like | 2.19 | 0.02375039 |
| CG10844-RC | Rya-r44F | muscle contraction; signaling | 3.15 | 0.00047735 |

| Flybase ID | name | function | M-value | p-value |
|---|---|---|---|---|
| *tissue structure* | | | | |
| CG18408-RI | CAP | focal adhesion | 1.02 | 0.00028029 |
| CG9540-RA | Antigen 5-related 2 | cellular component | 1.02 | 2.89E-05 |
| CG15884-RA | | structural constituent of chitin-based cuticle | 1.03 | 0.00012311 |
| CG10119-RA | Lamin C | structural molecule activity | 1.06 | 0.0080515 |
| CG8927-RA | | structural constituent of cuticle | 1.06 | 5.91E-05 |
| CG5154-RA | Imaginal disc growth factor 5 | chitin catabolic process | 1.08 | 0.00040236 |
| CG10533-RA | Lcp65Af | structural constituent of chitin-based cuticle | 1.10 | 0.525526 |
| CG14304-RA | | chitin metabolic process | 1.11 | 0.00688866 |
| CG7298-RA | | structural constituent of peritrophic membrane | 1.12 | 4.34E-05 |
| CG12008-RA | Kst | plasma membrane organization | 1.13 | 3.50E-05 |
| CG7216-RA | Acp1 | structural constituent of adult chitin-based cuticle | 1.17 | 0.4377084 |
| CG1869-RA | Cht7 | chitin catabolic process | 1.18 | 0.00206789 |
| CG8756-RB | Verm | chitin metabolic process | 1.19 | 0.00150816 |
| CG2555-RA | Cpr11B | structural constituent of chitin-based cuticle | 1.20 | 0.0073102 |
| CG31973-RA | Cda5 | chitin metabolic process | 1.22 | 0.00045718 |
| CG32304-RA | Obst-I | chitin metabolic process | 1.22 | 0.00023846 |
| CG2989-RA | Cth6 | chitin catabolic process | 1.25 | 0.00612129 |
| CG4367-RA | | chitin binding | 1.26 | 0.00079939 |
| CG7973-RA | Obst-H | chitin binding | 1.26 | 0.00036953 |
| CG7941-RA | Cpr67Fa1 | structural constituent of chitin-based cuticle | 1.27 | 0.20235803 |
| CG10287-RA | Gasp | structural constituent of peritrophic membrane | 1.28 | 0.00041367 |
| CG18349-RA | Cpr67Fa2 | structural constituent of chitin-based cuticle | 1.29 | 0.2309258 |
| CG7252-RA | | structural constituent of peritrophic membrane | 1.30 | 6.07E-05 |
| CG33468-RA | | cell adhesion | 1.30 | 0.02723392 |
| CG8779-RA | Neuromusculin | cell adhesion | 1.31 | 0.04613973 |
| CG18779-RA | | structural constituent of chitin-based cuticle | 1.32 | 0.36477137 |
| CG10534-RA | Lcp65Ag2 | structural constituent of chitin-based cuticle | 1.33 | 0.23168867 |
| CG32656-RA | | chitin binding | 1.42 | 2.71E-05 |
| CG32284-RA | | chitin binding protein | 1.43 | 0.0279217 |
| CG7306-RA | Obst-F | chitin metabolic process | 1.47 | 1.01E-05 |
| CG7874-RA | Mur18B | chitin metabolic process | 1.48 | 0.00033802 |
| CG6947-RA | | chitin binding protein; peritrophic matrix | 1.51 | 8.28E-05 |
| CG17824-RA | | chitin binding protein; peritrophic matrix | 1.54 | 0.00075799 |
| CG1919-RA | Cpr62Bc | cuticle protein; immune response | 1.56 | 0.00975316 |
| CG5883-RA | | chitin binding protein; peritrophic matrix | 1.56 | 3.72E-05 |
| CG31014-RA | Prolyl-4-hydroxylase-alpha SG1 | extracellular matrix formation | 1.62 | 0.0020444 |
| CG31439-RA | Muc96D | mucin; chitin binding | 1.67 | 0.00061142 |
| CG31015-RA | PH4αPV | prolyl hydroxylase; extracellular matrix formation | 1.71 | 0.00015381 |
| CG7356-RA | Tg | transglutaminase; hemolymph coagulation; cuticle formation | 1.77 | 0.00153428 |
| CG7876-RA | | chitin binding | 1.86 | 2.45E-05 |
| CG8515-RA | | cuticle protein; regulated by Trx and other chromatin regulators | 1.95 | 0.00613777 |
| CG2560-RA | Cpr11A | cuticle protein; regulated by Trx and other chromatin regulators | 1.99 | 0.01376928 |
| CG11142-RB | Obst-E | chitin binding, peritrophic matrix; cuticle forming tissue | 1.99 | 0.00060708 |

| Flybase ID | name | function | M-value | p-value |
|---|---|---|---|---|
| CG10725-RB | | chitin binding protein | 2.07 | 9.90E-05 |
| CG6452-RA | TweedleO | cuticle protein; regulated by Trx and other chromatin regulators | 2.12 | 0.3154996 |
| CG8695-RA | Larval visceral protein L | maltase; cuticle protein | 2.16 | 0.00285968 |
| CG14266-RA | New glue 2 | puparial adhesion; ecdysone target? | 2.37 | 0.00013305 |
| CG8510-RA | | insect cuticle protein | 2.38 | 0.00014529 |
| CG10781-RA | New glue 1 | puparial adhesion; ecdysone target? | 2.57 | 6.92E-05 |
| CG10154-RA | | chitin binding, peritrophic matrix; | 2.74 | 0.00080602 |
| CG5765-RA | Muc55B | epithelial surface; peritrophic matrix; gut-specific expression | 2.80 | 0.00025028 |
| CG15515-RA | | cuticle protein | 2.84 | 0.06527067 |
| CG4835-RA | | chitin binding protein | 3.34 | 1.80E-06 |
| CG13224-RA | | cuticle protein | 3.82 | 7.78E-05 |
| CG12491-RA | | structural protein? | 4.99 | 9.82E-07 |
| *transport* | | | | |
| CG16989-RA | | neurotransmitter transporter activity | 1.02 | 0.00079322 |
| CG7571-RA | Oatp74D | organic anion transmembrane transporter activity | 1.03 | 0.00054038 |
| CG5485-RA | Prestin | transmembrane transport | 1.04 | 0.01325953 |
| CG7458-RA | | transmembrane transport | 1.04 | 3.89E-05 |
| CG11147-RA | | ATPase activity, coupled to transmembrane movement of substances | 1.07 | 0.00039786 |
| CG1718-RA | | ATPase activity, coupled to transmembrane movement of substances | 1.09 | 0.00218354 |
| CG9702-RA | | transmembrane transport | 1.09 | 0.00168143 |
| CG11897-RA | | drug transmembrane transporter activity | 1.10 | 0.00515093 |
| CG5326-RA | | transmembrane transport | 1.11 | 0.00048493 |
| CG7084-RB | | transmembrane transport | 1.11 | 0.00048306 |
| CG6214-RB | MRP | ATPase activity, coupled to transmembrane movement of substances | 1.11 | 1.31E-05 |
| CG6901-RA | | transmembrane transport | 1.12 | 0.00457337 |
| CG8028-RA | | transmembrane transport | 1.12 | 0.00211748 |
| CG9413-RA | | amino acid transmembrane transport | 1.14 | 0.00627118 |
| CG10181-RA | Multiple drug resistance 65 | drug transmembrane transporter activity | 1.18 | 0.00083346 |
| CG11209-RA | Pickpocket 6 | sodium ion transport | 1.26 | 0.03573231 |
| CG33105-RA | P24-related-2 | transport | 1.26 | 0.03923185 |
| CG31792-RA | | ATPase activity, coupled to transmembrane movement of substances | 1.28 | 0.0002119 |
| CG7678-RA | Vha100-4 | ATP synthesis coupled proton transport | 1.28 | 0.0003122 |
| CG30016-RA | | transport | 1.32 | 2.27E-05 |
| CG12787-RC | Hoepel 1 | transmembrane transport | 1.34 | 0.0003823 |
| CG6186-RB | Transferrin 1 | iron ion transmembrane transporter activity | 1.36 | 0.00348289 |
| CG31743-RA | | sulfotransferase activity | 1.38 | 0.00014004 |
| CG3424-RA | Pathetic | amino acid transporter | 1.38 | 1.05E-05 |
| CG12251-RA | AQP | water transmembrane transporter activity | 1.40 | 0.0006666 |
| CG2191-RA | Smvt | sodium-dependent multivitamin transmembrane transporter activity | 1.42 | 0.04896085 |
| CG6186-RA | Transferrin 1 | iron ion transmembrane transporter activity | 1.43 | 0.00159803 |
| CG10505-RA | | ATPase activity, coupled to transmembrane movement of substances | 1.44 | 0.00259139 |
| CG3380-RA | Oatp58Dc | organic anion transmembrane transporter activity | 1.45 | 0.00052837 |
| CG9981-RA | | ATPase activity, coupled to transmembrane movement of ions | 1.47 | 0.01852656 |
| CG5685-RA | Na/Ca-exchange protein | calcium:sodium antiporter activity | 1.49 | 0.00031508 |

| Flybase ID | name | function | M-value | p-value |
|---|---|---|---|---|
| CG13124-RA | GRASP65 | Golgi component | 1.54 | 0.00023469 |
| CG9994-RA | Rab9 | retrograde transport, endosome to Golgi | 1.55 | 0.00020887 |
| CG17636-RA | | gamma glutamyltranspeptidase | 1.57 | 0.00065338 |
| CG15407-RB | | sugar transporter | 1.59 | 0.00028527 |
| CG7499-RA | Rh50 | ammonium transporter | 1.59 | 0.00290443 |
| CG30272-RA | | major facilitator subfamily | 1.61 | 0.00511953 |
| CG6417-RA | Oatp33 | organic anion transport | 1.65 | 1.10E-05 |
| CG7912-RA | | sulphate transporter | 1.66 | 0.00184297 |
| CG8791-RA | | transmembrane transport (major facilitator family) | 1.68 | 0.00466724 |
| CG2196-RA | Salty dog | sodium solute symporter; salt stress response | 1.71 | 0.00081837 |
| CG8051-RA | | transmembrane transport (major facilitator family) | 2.01 | 0.00027006 |
| CG2187-RA | | sodium solute symporter | 2.02 | 1.52E-05 |
| CG4314-RA | Scarlet | ABC transporter | 2.02 | 0.01022289 |
| CG4830-RA | | long-chain fatty acid transporter activity | 2.12 | 0.00047681 |
| CG6293-RA | | L-ascorbate:sodium symporter activity | 2.32 | 0.00018091 |
| CG9780-RA | | ABC transporter | 2.36 | 0.00492502 |
| CG18327-RA | | mitochondrial transporter | 2.50 | 0.00188167 |
| CG31052-RA | Na+/H+ hydrogen antiporter 2 | transporter | 2.61 | 3.30E-05 |
| CG4991-RB | | amino acid transporter | 2.77 | 0.00028218 |
| CG9825-RA | | transmembrane transport (major facilitator family) | 2.80 | 0.00057275 |
| CG8654-RA | | transmembrane transport (major facilitator family) | 3.30 | 0.00012308 |
| *unknown function* | | | | |
| CG9682-RA | | unknown function | 1.00 | 0.00010784 |
| CG11158-RA | | unknown function | 1.01 | 1.70E-05 |
| CG14059-RA | | unknown function | 1.01 | 0.01854762 |
| CG17147-RA | | unknown function | 1.01 | 8.15E-05 |
| CG7992-RA | | unknown function | 1.02 | 0.00013101 |
| CG15597-RA | | unknown function | 1.02 | 0.18330239 |
| CG13360-RA | | unknown function | 1.03 | 0.00501462 |
| CG15236-RB | | unknown function | 1.03 | 0.00026727 |
| CG8486-RA | | unknown function | 1.03 | 0.00226846 |
| CG4786-RA | | unknown function | 1.03 | 5.41E-06 |
| CG32485-RA | | unknown function | 1.03 | 0.0014073 |
| CG11168-RA | | unknown function | 1.04 | 0.00045324 |
| CG4405-RB | Junctophilin | unknown function | 1.04 | 8.92E-05 |
| CG15523-RA | | unknown function | 1.04 | 2.53E-05 |
| CG5399-RA | | unknown function | 1.04 | 0.0001821 |
| CG5819-RA | | unknown function | 1.05 | 1.11E-05 |
| CG15043-RA | | unknown function | 1.05 | 0.00955917 |
| CG8177-RC | | unknown function | 1.06 | 0.00159165 |
| CG8087-RA | | unknown function | 1.06 | 0.11410831 |
| CG31321-RB | | unknown function | 1.07 | 0.0124758 |
| CG12075-RA | | unknown function | 1.07 | 0.02159198 |
| CG7920-RB | | unknown function | 1.07 | 0.00054043 |

| Flybase ID | name | function | M-value | p-value |
|---|---|---|---|---|
| CG14907-RA | | unknown function | 1.07 | 0.00018496 |
| CG33075-RA | | unknown function | 1.07 | 0.00165134 |
| CG12826-RA | | unknown function | 1.07 | 0.00177339 |
| CG5506-RA | | unknown function | 1.07 | 0.00298224 |
| LP09564 | | unknown function | 1.07 | 0.0020261 |
| CG18594-RA | | unknown function | 1.08 | 0.00825649 |
| CG32714-RA | | unknown function | 1.09 | 0.00235881 |
| CG10936-RB | | unknown function | 1.10 | 0.0105172 |
| CG13806-RA | | unknown function | 1.10 | 0.00017254 |
| CG6129-RB | | unknown function | 1.11 | 0.00228191 |
| CG5084-RA | | unknown function | 1.11 | 1.70E-05 |
| GM03661 | | unknown function | 1.11 | 0.00154119 |
| CG14456-RA | | unknown function | 1.12 | 0.00112723 |
| CG6579-RA | | unknown function | 1.12 | 3.57E-05 |
| CG2010-RB | | unknown function | 1.14 | 0.01799297 |
| CG4446-RA | | unknown function | 1.15 | 0.00144065 |
| CG5767-RA | | unknown function | 1.15 | 2.08E-05 |
| CG8852-RA | | unknown function | 1.16 | 0.00270663 |
| CG31974-RA | | unknown function | 1.16 | 0.00398821 |
| CG40198-RA | | unknown function | 1.16 | 0.49518213 |
| CG12177-RA | | unknown function | 1.17 | 0.00068612 |
| CG6327-RA | | unknown function | 1.18 | 0.00054308 |
| CG13912-RA | | unknown function | 1.18 | 0.00439764 |
| CG13003-RA | | unknown function | 1.19 | 0.00995412 |
| CG7915-RA | | unknown function | 1.19 | 9.90E-05 |
| CG11313-RA | | unknown function | 1.20 | 0.00117956 |
| CG14273-RA | | unknown function | 1.20 | 4.78E-05 |
| CG32603-RA | | unknown function | 1.20 | 0.07317999 |
| CG5866-RA | | unknown function | 1.21 | 0.06991144 |
| CG8986-RA | | unknown function | 1.21 | 0.00560093 |
| CG31769-RA | | unknown function | 1.22 | 0.00071505 |
| CG4733-RA | | unknown function | 1.22 | 2.34E-05 |
| CG1819-RA | | unknown function | 1.23 | 0.00078614 |
| CG6023-RA | | unknown function | 1.23 | 0.01324493 |
| CG32352-RB | | unknown function | 1.23 | 0.00032724 |
| CG2157-RA | | unknown function | 1.23 | 0.00196705 |
| CG9399-RB | | unknown function | 1.24 | 0.00224034 |
| CG30411-RA | | unknown function | 1.24 | 0.0017123 |
| CG9568-RA | | unknown function | 1.25 | 0.00163339 |
| CG31446-RA | | unknown function | 1.25 | 0.00342616 |
| CG18635-RA | | unknown function | 1.25 | 0.00872186 |
| CG1583-RA | | unknown function | 1.26 | 0.00015071 |
| CG1698-RA | | unknown function | 1.26 | 0.00083166 |
| CG1961-RA | | unknown function | 1.27 | 3.82E-05 |
| CG15199-RA | | unknown function | 1.27 | 0.00071054 |

| Flybase ID | name | function | M-value | p-value |
|---|---|---|---|---|
| CG31086-RA | | unknown function | 1.27 | 0.0065562 |
| CG9626-RA | | unknown function | 1.30 | 0.00023529 |
| CG31259-RA | | unknown function | 1.31 | 0.00282538 |
| CG17108-RA | | unknown function | 1.31 | 0.00606994 |
| CG14893-RA | | unknown function | 1.32 | 0.00824062 |
| CG5535-RA | | unknown function | 1.33 | 0.0035108 |
| CG31269-RA | | unknown function | 1.36 | 0.02612082 |
| CG33469-RA | | unknown function | 1.37 | 0.00017706 |
| CG13323-RA | | unknown function | 1.37 | 0.00472046 |
| CG16820-RA | | unknown function | 1.38 | 0.00306586 |
| CG14033-RA | | unknown function | 1.38 | 0.00325556 |
| CG31464-RA | | unknown function | 1.40 | 0.00026532 |
| HDC07637 | | unknown function | 1.40 | 0.00193492 |
| CG14398-RA | | unknown function | 1.41 | 0.00318984 |
| CG10121-RC | | unknown function | 1.44 | 0.00041633 |
| CG16777-RA | | unknown function | 1.44 | 0.00271698 |
| CG7900-RA | | unknown function | 1.44 | 0.00696215 |
| CG11915-RA | | unknown function | 1.45 | 9.42E-06 |
| CG31288-RA | | unknown function | 1.46 | 0.01209879 |
| CG40146-RA | | unknown function | 1.46 | 0.00102306 |
| CG12105-RA | | unknown function | 1.46 | 0.00549115 |
| CG32922-RA | | unknown function | 1.46 | 0.00135565 |
| CG3884-RA | | unknown function | 1.46 | 0.00013103 |
| CG13492-RA | | unknown function | 1.47 | 0.002014 |
| CG14616-RD | Lethal (1) G0196 | unknown function | 1.48 | 0.00052125 |
| HDC03517 | | unknown function | 1.48 | 0.05001477 |
| CG33205-RC | | unknown function | 1.48 | 0.00164237 |
| CT22789 | | unknown function | 1.49 | 0.00521984 |
| CG7549-RA | | unknown function | 1.50 | 0.00709325 |
| CG31781-RA | | unknown function | 1.51 | 0.00167286 |
| CG15412-RA | | unknown function | 1.51 | 3.97E-05 |
| CG11160-RB | | unknown function | 1.52 | 0.00123941 |
| CG10597-RA | | unknown function | 1.52 | 0.09280681 |
| CG15741-RA | | unknown function | 1.54 | 0.32496153 |
| CG10732-RB | | unknown function | 1.55 | 0.00123446 |
| CG7631-RA | | unknown function | 1.58 | 0.01126819 |
| CG32198-RB | | unknown function | 1.64 | 0.00022207 |
| CG15213-RA | | unknown function | 1.64 | 0.00054515 |
| CG7300-RB | | unknown function | 1.66 | 0.03025559 |
| CG31531-RA | | unknown function | 1.68 | 9.48E-05 |
| CG14852-RA | | unknown function | 1.68 | 0.04999475 |
| CG34278-RA | | unknown function | 1.70 | 0.0030693 |
| CG13102-RA | | unknown function | 1.73 | 0.00020561 |
| CG9077-RA | | unknown function | 1.73 | 0.02664439 |
| CG31558-RA | | unknown function | 1.73 | 0.00132833 |

| Flybase ID | name | function | M-value | p-value |
|---|---|---|---|---|
| CG31781-RB | | unknown function | 1.74 | 0.00103567 |
| GM10545 | | unknown function | 1.74 | 0.00836537 |
| HDC03535 | | unknown function | 1.75 | 7.78E-05 |
| CG15530-RA | | unknown function | 1.77 | 0.00047886 |
| CG9396-RA | | unknown function | 1.82 | 0.00854715 |
| CT37020 | | unknown function | 1.83 | 0.00781421 |
| CG4371-RA | | unknown function | 1.85 | 0.13133808 |
| CG13705-RA | | unknown function | 1.92 | 0.25265583 |
| CG13946-RA | | unknown function | 1.97 | 0.00587546 |
| CG4962-RA | | unknown function | 2.01 | 0.00033276 |
| CG14292-RA | | unknown function | 2.04 | 0.00019122 |
| CG13512-RA | | unknown function | 2.16 | 9.33E-06 |
| CG32241-RA | | unknown function | 2.24 | 0.00203155 |
| CG5810-RA | | unknown function | 2.26 | 1.98E-06 |
| CG13460-RA | | unknown function | 2.26 | 3.55E-05 |
| CG18649-RA | | unknown function | 2.34 | 0.00040003 |
| CG11350-RB | | unknown function | 2.40 | 0.07460317 |
| CG8773-RA | | unknown function | 2.42 | 0.00043929 |
| CG32355-RA | | unknown function | 2.48 | 0.00091557 |
| CG2064-RA | | unknown function | 2.55 | 7.58E-06 |
| CG14120-RA | | unknown function | 2.59 | 0.00028705 |
| CG14879-RA | | unknown function | 2.62 | 4.69E-06 |
| CG32073-RA | | unknown function | 2.63 | 0.01714194 |
| CG10953-RA | | unknown function | 2.64 | 0.00479745 |
| CG9555-RA | | unknown function | 3.00 | 2.28E-05 |
| CG10843-RA | | unknown function | 3.13 | 0.00738902 |
| CG12506-RA | | unknown function | 3.31 | 0.00020776 |
| CG32564-RA | | unknown function | 3.90 | 0.00885644 |
| CG8534-RA | | unknown function | 1.63 | 0.04355153 |
| CG13640-RA | | unknown function | 2.64 | 0.00064365 |
