# Supplementary material for: CHD1 Contributes to Intestinal Resistance against Infection by P. aeruginosa in Drosophila melanogaster
Source: PLoS One. 2012 Aug 13;7(8):e43144. doi: 10.1371/journal.pone.0043144 (PMC3418260; doi:10.1371/journal.pone.0043144)
Supplement: Table S2 — Downregulated genes in Chd1−/− flies. (PDF) [file pone.0043144.s008.pdf]

Flybase ID	name	function	M-value	p-value
detoxification				
CG5137-RA	Cyp312a1	monooxygenase activity	-1.01	0.001030014
CG3050-RB	Cyp6d5	oxidation reduction	-1.03	1.40E-05
CG9964-RA	Cyp309a1	oxidation reduction (myc target)	-1.17	0.0002673
CG17524-RA	Glutathione S transferase E3	glutathione transferase activity	-1.21	3.62E-05
CG18548-RA	Glutathione S transferase D10	glutathione transferase activity	-1.29	0.032076054
CG32041-RB		stress response	-1.55	0.046828202
CG1702-RA		glutathione transferase activity	-1.57	0.000700857
CG6762-RA		sulfiredoxin	-1.66	0.000325529
CG4183-RA	Heat shock protein 26	stress response, life span	-1.72	0.005368553
CG5164-RA	Glutathione S transferase E1	glutathione transferase activity	-1.72	5.41E-06
CG33192-RA	Metallothionein D	detoxification, immune response	-1.80	9.87E-05
CG17323-RA		UDP glucosyltransferase; Detoxification	-1.88	5.73E-05
CG5097-RA	Metallothionein C	detoxification	-2.01	0.004224879
CG4181-RA	Glutathione S transferase D2	glutathione transferase activity	-2.09	0.079766165
CG11512-RA	Glutathione S transferase D4	detoxification	-2.21	0.045854333
CG4190-RA	Heat shock gene 67Bc	stress response	-2.32	4.04E-05
CG33126-RA	Neural Lazarillo	Lipocalin-like; stress response	-2.55	4.34E-05
CG7227-RA		scavenger receptor	-2.81	2.55E-05
CG4312-RA	Metallothionein B	detoxification; gut-specific expression	-3.45	0.000648698
development				
CG9891-RA	Yellow-d2	involved in wing differentiation?	-1.01	0.001280823
CG14728-RA	Shadow	ecdysone biosynthesis	-1.02	0.010684775
CG15151-RA	Pray For Elves	ocellus development	-1.08	0.001896923
CG32356-RB	Ecdysone-inducible gene E1	imaginal disc eversion	-1.20	0.041729981
CG16993-RA	Inturned	establishment of planar polarity	-1.21	0.003249892
CG7802-RA		regulation of embryonic cell shape	-1.29	0.001334525
CG15153-RA		developmental role?; protease?	-1.47	0.013389789
CG18607-RA		pupation?	-1.54	4.08E-06
CG40050-RA		ecdysone biosynthesis	-1.68	0.00063217
CG9005-RA		mesoderm development?	-1.88	4.94E-05
CG32356-RA	Ecdysone-inducible gene E1	ecdysone induced gene	-1.92	0.000356763
CG2016-RB	CG2016	juvenile hormone binding	-2.41	3.16E-05
CG7350-RA	Ecdysone-induced gene 71Ed	ecdysone induced gene	-2.44	0.053428096
CG11129-RA	Yolk protein 3	embryonic development	-2.47	0.046754052
CG32180-RA	Ecdysone-induced protein 74EF	ecdysone induced protein	-2.51	0.000393598
CG2723-RA	Ecdysone-inducible gene E3	ecdysone induced gene	-2.55	9.63E-05
CG18023-RA	Ecdysone-induced protein 78C	ecdysone induced protein; nuclear receptor	-2.80	0.000182518
CG1934-RA	Ecdysone-inducible gene E2	ecdysone induced gene	-2.92	0.000136768
CG16931-RA	Ecdysone-induced gene 71Ea	ecdysone induced gene	-2.99	0.010101387
CG7608-RA	Ecdysone-induced gene 71Ec	ecdysone induced gene	-3.12	0.037970312
CG4354-RA	Slow border cells	transcription factor; cell migration; JAK/STAT pathway	-3.12	3.65E-05

Flybase ID	name	function	M-value	p-value
CG7336-RA	Ecdysone-induced gene 71Eg	ecdysone induced gene	-3.28	0.004077863
CG7355-RA	Ecdysone-induced gene 71Eb	ecdysone induced gene	-5.46	0.016702952
immune response				
CG32858-RC	Singed	wound healing	-1.07	0.000187664
CG4466-RA	Heat shock protein 27	defense response	-1.13	0.001244906
CG10810-RA	Drosomycin	antibacterial humoral response	-1.15	0.020147264
CG8175-RA	Metchnikowin	defense response	-1.19	0.028932148
CG8157-RA		immune response?	-1.42	2.34E-06
CG4091-RA		caspase inhibitor; saliv. gland autophagic cell death; gut-specific	-1.50	4.79E-07
CG9120-RA	Lysozyme X	immune response	-1.54	0.000245
CG10146-RA	Attacin-A	antimicrobial peptide; immune response	-1.57	0.026950148
CG8337-RA	E(spl) region transcript malpha	Notch signaling pathway; intestinal stem cell maintenance	-1.58	0.001553038
CG7104-RA	Spz3	Toll pathway	-1.59	0.000981998
CG4471-RA	Tetraspanin 42Ep	immune response; growth factor signalling; cell adherence	-1.59	0.00065319
CG5489-RB	Autophagy-specific gene 7	autophagy; midgut-specific; immune response	-1.71	0.000176281
CG1887-RA		scavenger receptor; defense response	-1.73	0.003786299
CG6104-RA	E(spl) region transcript m2	Notch signaling pathway; stem cell maintenance in the intestine	-1.78	0.000207526
CG9616-RA		immunity related?	-1.79	0.000698169
CG18146-RA		phagocytosis receptor	-1.80	0.000840605
CG2056-RB		serine protease; immune response, required for Toll activation	-1.91	0.000792246
CG4859-RB	Matrix metalloproteinase 1	matrix metalloproteinase; autophagy; wound healing response	-1.92	0.000110447
CG7225-RA	Windbeutel	thioredoxin fold; Toll signaling pathway	-1.94	0.000237634
CG31410-RA		lipid recognition- pathogen response?	-2.10	0.000675385
CG14745-RA	PGRP-SC2	peptidoglycan binding protein; negative regulator of Imd pathway	-2.17	7.19E-05
CG12789-RA		scavenger receptor; defense response	-2.20	8.17E-06
CG18106-RA	Immune induced molecule 2	defense response	-2.20	0.000880537
CG11425-RA		phosphatidate phosphatase; immune response?	-2.45	5.58E-07
CG2065-RA		oxidoreductase; gut-specific immune response; oxidative stress	-2.62	0.00166409
CG3397-RA		oxidoreductase; JAK/STAT connection?	-2.63	0.003032741
CG12172-RA	Serine protease inhibitor 43Aa	serine protease inhibitor (Serpin); immune response	-2.64	2.66E-05
CG6429-RA		regulated by immune response; destabilase motif	-2.77	0.0003151
CG16844-RA		defense response; antibacterial humoral response	-2.85	0.001207914
CG9681-RA	PGRP-SB1	peptidoglycan binding protein; negative regulator of Imd pathway	-3.21	0.00400283
CG18108-RA	Immune induced molecule 1	defense response	-3.27	7.50E-05
CG10812-RA	Drosomycin-5	antimicrobial peptide; immune response	-3.45	0.001539475
CG1342-RA		protease inhibitor; development; immune function	-3.91	0.005733107
CG13606-RA		antiviral response	-4.58	0.032568116
CG9697-RA	PGRP-SB2	peptidoglycan recognition protein; Imd pathway	-5.44	0.017175862
CG32279-RA		defense response to fungus	-5.65	0.005754346
intracellular processes				
CG5745-RA		phagocytosis, engulfment	-1.01	0.000622362
CG17754-RC		phagocytosis, engulfment	-1.03	0.000764452

Flybase ID	name	function	M-value	p-value
CG11312-RA	Inscuteable	cytoskeletal adaptor activity	-1.03	0.000996994
CG8420-RA		endoplasmic reticulum	-1.03	0.000451986
CG12023-RB	GV1	DNA binding	-1.04	0.000346491
CG9871-RA	RpL22-like	translation	-1.05	0.007024217
CG5338-RB	Ribosomal protein S19b	translation	-1.05	0.037440395
CG17949-RA		selective histone variant exchange at DNA lesions?	-1.07	0.026494009
CG4496-RA		nucleic acid binding	-1.07	0.01714467
CG2374-RA	Late bloomer	synapse assembly	-1.07	0.006820408
CG3875-RA		RNA binding	-1.07	0.049785939
CG7266-RC	Eip71CD	autophagic cell death; salivary gland cell autophagic cell death	-1.08	0.000118678
CG8364-RB	Drep-3	apoptosis	-1.09	2.22E-05
CG32971-RA		regulation of transcription from RNA polymerase II promoter	-1.09	0.054978572
CG13597-RA		nuclear mRNA splicing, via spliceosome	-1.09	0.028773837
CG1030-RA	Sex combs reduced	sequence-specific DNA binding transcription factor activity	-1.10	0.002226851
CG3827-RA	Scute	specific RNA polymerase II transcription factor activity	-1.12	9.10E-05
CG5134-RC	Mediator complex subunit 9	RNA polymerase II transcription mediator activity	-1.13	0.001686233
CG30431-RA		Zn finger	-1.13	2.24E-05
CG9015-RB	Engrailed	specific RNA polymerase II transcription factor activity	-1.14	0.000849058
CG17450-RB		microtubule cytoskeleton organization	-1.14	0.051576733
CG3726-RA		DNA binding	-1.16	0.003632165
CG5632-RA	Thoc6	mRNA export from nucleus	-1.16	0.001247251
CG9873-RA	Ribosomal protein L37b	translation	-1.17	0.032944181
CG32171-RB	Limpet	transcription factor activity (Txn factor); wing development	-1.17	0.004278419
CG31642-RA		Zn finger; cell cycle regulation?	-1.20	0.023097065
CG4167-RA	Heat shock gene 67Ba	response to heat	-1.22	8.55E-05
CG6829-RB	Apaf-1-related-killer	apoptosis	-1.26	0.003622304
CG31274-RA		protein binding (leucin rich repeat)	-1.26	0.001123412
CG18313-RA		nascent polypeptide-associated complex NAC	-1.27	0.000281608
CG6891-RA		actin binding	-1.28	0.00116776
CG12493-RA		dsRNA-binding	-1.30	0.049934007
CG12023-RA	GV1	DNA binding	-1.33	7.72E-05
CG18446-RA		zinc ion binding (zinc finger)	-1.34	0.002560575
CG8335-RA		initiation of translation	-1.35	0.005346831
CG31617-RA	Histone H1	chromatin assembly or disassembly	-1.37	0.000359777
CG18389-RA	Eip93F	sequence-specific DNA binding transcription factor activity	-1.38	0.002510417
CG11386-RA		ribosomal protein?	-1.39	0.114254519
CG11186-RA	Twin of eyeless	sequence-specific DNA binding transcription factor activity	-1.40	0.002830151
CG8361-RA	E(spl) region transcript m7	sequence-specific DNA binding transcription factor activity	-1.41	0.001162579
CG4080-RA		zinc ion binding (RING type zi finger); developmental regulation?	-1.44	8.34E-05
CG15632-RA	Taf12L	transcription initiation from RNA polymerase II promoter	-1.44	0.029461149
CG32601-RA		regulation of transcription	-1.44	0.022561282
CG12477-RA		protein binding; zinc ion binding (zink finger protein)	-1.49	0.012491481
CG8679-RA		lamin?	-1.54	0.007060087
CG8365-RA	Enhancer of split	transcription factor; Notch signaling pathway	-1.57	0.000506968

Flybase ID	name	function	M-value	p-value
CG11186-RB	twin of eyeless	transcription factor	-1.59	0.001823692
CG31618-RA	Histone H2A	nucleosome assembly; chromatin assembly or disassembly	-1.60	0.005730474
CG10630-RA		dsRNA-binding	-1.64	0.021409548
CG4021-RA		RNA-binding	-1.83	0.010655295
CG7804-RA		RNA-binding	-1.89	0.005326745
CG9989-RA		nuclease; stress response ?	-1.94	0.000689577
CG17763-RA		PcG target?	-2.32	0.001319308
CG17386-RA		putative transcription factor; circadian regulation	-3.53	0.003024423
CG3733-RA	Chd1	chromatin remodeling	-6.24	3.22E-08
metabolism				
CG7820-RA	Carbonic anhydrase 1	carbonate dehydratase activity	-1.02	2.02E-05
CG14716-RA	Heme oxygenase	heme oxygenase (decyclizing) activity	-1.02	0.000577751
CG1637-RA		acid phosphatase activity	-1.04	0.000410626
CG1941-RA		diacylglycerol acyltransferase	-1.04	0.013186072
CG7742-RA		Rab GTPase activator activity	-1.04	0.135733132
CG3376-RB		sphingomyelin phosphodiesterase activity	-1.04	0.007878029
CG4774-RB		phospholipid biosynthesis	-1.07	3.25E-05
CG1443-RA		oxidation-reduction process	-1.09	0.005092746
CG5554-RA		protein disulfide isomerase activity	-1.11	0.000546284
CG2916-RB	Sep5	GTPase activity	-1.14	0.003018564
CG14904-RA	Scp2	GTPase activity	-1.15	9.47E-05
CG4551-RA	Smell impaired 35A	protein serine/threonine kinase activity	-1.16	0.001532996
CG31628-RA	Adenosine 3	purine base biosynthetic process	-1.17	0.000754997
CG5854-RA		cellular metabolic process	-1.18	0.000646675
CG1942-RA		diacylglycerol acyltransferase	-1.23	0.016773922
CG6957-RA	Oscillin	glucosamine-6-phosphate deaminase activity	-1.23	0.000417861
CG15343-RA		pyridoxin biosynthesis	-1.24	0.002155161
CG18528-RA		GTPase activity	-1.24	6.68E-05
CG4546-RA		arginine kinase activity	-1.26	0.024832441
CG9509-RA		alcohol metabolic process	-1.27	9.06E-05
CG8565-RA		protein amino acid phosphorylation (pre-mRNA processing)	-1.30	0.038460016
CG13833-RA		oxidation-reduction process	-1.33	0.009874203
CG8112-RA		sterol O-acyltransferase activity	-1.34	0.001325882
CG3264-RA		alkaline phosphatase activity	-1.35	0.034203055
CG4665-RA	Dihydropteridine reductase	6,7-dihydropteridine reductase activity	-1.37	0.001807162
CG6287-RA		phosphoglycerate dehydrogenase activity	-1.39	4.85E-05
CG11170-RB		ATPase activity	-1.40	0.000948726
CG7447-RA	Slow	calcium ion binding	-1.41	4.05E-05
CG4586-RA		beta-oxidation	-1.41	8.74E-05
CG17191-RA		lipid metabolic process (phospholipase A1 similarity)	-1.41	0.002875555
CG12539-RA		glucose dehydrogenase activity	-1.42	0.007742095
CG6818-RA	MESR6	metal ion binding	-1.47	0.000131243
CG33096-RB		phospholipase	-1.47	0.000705699

Flybase ID	name	function	M-value	p-value
CG18550-RA	Yellow-f	melanin biosynthetic process from tyrosine	-1.48	0.002109513
CG9441-RB	Punch	GTP cyclohydrolase I activity	-1.49	0.000159265
CG3940-RA		carbonic anhydrase	-1.49	0.001798907
CG7320-RA		oxygen transport; hemocyanine	-1.52	0.054924638
CG17148-RA	Esterase P	esterase activity	-1.55	0.000111836
CG8100-RA		oxygen transport	-1.56	0.000218446
CG1851-RA	Ady43A	adenosine kinase	-1.56	0.00108971
CG18466-RB	Nmdmc	tetrahydrofolate reductase	-1.60	0.000223127
CG14946-RA		oxidoreductase	-1.69	0.000776708
CG8421-RE	Aspartyl beta-hydroxylase	aspartyl β-hydroxylase	-1.70	0.001630955
CG8782-RA	Oat	ornithine aminotransferase precursor	-1.71	4.74E-06
CG9486-RA		GCN5-related N-acetyltransferase	-1.76	0.0026171
CG4842-RA		oxidoreductase	-1.79	0.001775652
CG40123-RA		chitin-based cuticle; ecdysone biosynthetic process	-1.79	0.000627353
CG10697-RB	Dopa decarboxylase	dopa decarboxylase; neurotransmitter metabolism; pigmentation	-1.95	0.000761067
CG5618-RA		pyridoxal phosphatase	-1.99	1.90E-05
CG10852-RA	Accessory gland peptide 63F	accessory gland peptide; protease inhibitor'?	-2.08	0.00011795
CG10178-RA		glucuronosyltransferase; molting block?	-2.49	0.000116082
CG5171-RB		trehalose phosphatase	-2.75	1.22E-05
CG4182-RA	Yellow-c	metabolic function	-2.78	0.000137875
CG4382-RA		carboxyl esterase domain	-2.93	1.88E-08
CG8093-RA		lipase activity; gut specific expression; immune response?	-3.14	0.000596973
CG6277-RA		lipid metabolism; gut-specific expression; immune response?	-3.22	0.001630936
CG2759-RA	White		-3.67	8.25E-06
CG9452-RA		histidine phosphatase	-3.78	0.002372192
CG18606-RA		Acyl-CoA N-acetyltransferase domain	-3.86	3.31E-05
CG14406-RA		oxidoreductase	-4.04	3.18E-05
CG2070-RA		oxidoreductase	-4.40	0.022878436
CG16733-RA		sulfotransferase; retinol dehytratase similarity	-4.47	1.98E-06
CG3331-RA	Ebony	neurotransmitter metabolism; melanin synthesis pathway	-4.56	0.083010064
proteolysis				
CG4637-RA	Hedgehog	cysteine-type endopeptidase activity	-1.01	0.001662671
CG31778-RA		serine-type endopeptidase inhibitor activity	-1.05	0.000674879
CG32063-RB		aminopeptidase activity	-1.13	0.041208804
CG2241-RA	Rpt6R	proteolysis	-1.15	0.002626721
CG6687-RA	Spn88Eb	serine-type endopeptidase inhibitor activity	-1.15	0.000116644
CG32351-RA		aminopeptidase activity	-1.15	0.047667445
CG8586-RA		serine-type endopeptidase activity	-1.19	0.0028388
CG4316-RA	Stubble	serine-type endopeptidase activity	-1.21	0.000866775
CG9456-RA	Serine protease inhibitor 1	serine-type endopeptidase inhibitor activity	-1.23	0.001259643
CG11023-RA		cysteine-type endopeptidase activity	-1.25	0.024682804
CG3066-RA	Serine protease 7	serine-type carboxypeptidase activity	-1.32	0.000723864
CG10764-RA		serine-type endopeptidase activity	-1.36	0.000321714

Flybase ID	name	function	M-value	p-value
CG31039-RA	Jonah 99Ci	serine-type endopeptidase; digestion	-1.41	0.000560116
CG4408-RA		metallocarboxypeptidase activity	-1.42	0.000455056
CG8550-RA		metalloendopeptidase activity	-1.47	0.00049128
CG30289-RA		serine-type endopeptidase activity	-1.50	0.00065761
CG11169-RA		metalloprotease; hormone induced	-1.58	0.002929072
CG31777-RA		protease inhibitor	-1.63	0.001255606
CG4914-RA		serine protease	-1.63	1.55E-05
CG30288-RA		serine-type endopeptidase activity	-1.68	4.10E-05
CG9850-RA		metalloprotease	-1.72	0.002346063
CG9761-RA	Neprilysin 2	metalloprotease	-1.82	0.000658755
CG32627-RA		metalloprotease	-1.91	2.76E-06
CG11529-RA		serine-type endopeptidase activity	-1.97	0.000236411
CG3355-RA		serine-type endopeptidase activity	-1.99	0.00063296
CG30098-RA		serine-type endopeptidase activity	-2.00	5.06E-05
CG11956-RA	SP1029	metalloprotease	-2.32	0.000219511
CG5470-RA		serine-type endopeptidase activity	-2.33	0.001378
CG30091-RA		serine-type endopeptidase activity	-2.42	6.52E-05
CG4650-RA		serine-type endopeptidase activity	-2.53	0.000684786
CG1773-RA		endopeptidase	-2.57	6.99E-05
CG3700-RA		serine-type endopeptidase activity	-2.67	6.43E-06
CG10073-RA		peptidase activity	-2.70	0.001205037
CG3502-RA		metalloprotease	-2.71	0.004661248
CG3097-RA		metalloprotease	-2.75	8.41E-05
CG7906-RA		protease inhibitor; development?	-2.85	0.010688096
CG13748-RA		protease inhibitor; development?	-2.91	0.017382895
CG7924-RA		protease inhibitor; development?	-3.71	0.040508899
CG32762-RA		metalloprotease	-3.89	0.004437948
CG11459-RA		cysteine-type endopeptidase activity	-4.01	0.000187592
CG12951-RA		serine-type endopeptidase activity	-4.02	6.03E-05
signaling				
CG4722-RA	Big brain	Notch signaling pathway	-1.02	0.000451475
CG18024-RA	SoxNeuro	regulation of Wnt receptor signaling pathway	-1.05	0.000416919
CG6965-RA	Methuselah-like 5	G-protein coupled receptor protein signaling pathway	-1.07	0.000515417
CG6099-RA	E(spl) region transcript m4	Notch signaling pathway	-1.09	0.00027273
CG17760-RA		G-protein coupled receptor protein signaling pathway	-1.28	0.014294238
CG31421-RA	Tak1-like 1	MAP kinase kinase kinase activity	-1.45	0.008029943
CG6908-RA		kinase like	-1.61	9.75E-05
CG1916-RA	Wnt oncogene analog 2	WNT growth factor; Wnt signaling	-1.72	2.42E-08
CG8238-RA	Buffy	apoptosis regulator; Bcl2-like	-1.75	0.000431393
CG9259-RA		kinase-like	-1.99	6.19E-05
CG32447-RA		membrane receptor	-2.13	0.00072117
CG31104-RA		kinase-like	-2.91	4.39E-05
CG31557-RA	Odorant-binding protein 83ef	sensory perception of chemical stimulus	-3.98	0.00066687

Flybase ID	name	function	M-value	p-value
CG11893-RA		kinase like, interacts with bicoid interacting protein	-4.48	8.42E-05
tissue structure				
CG10112-RA	Cpr51A	structural constituent of chitin-based cuticle	-1.04	0.000785107
CG15757-RA	Cpr12A	structural constituent of chitin-based cuticle	-1.04	0.003120476
CG4383-RA		plasma membrane	-1.07	0.00215372
CG3440-RA	Pupal cuticle protein	structural constituent of pupal chitin-based cuticle	-1.11	0.002371178
CG12009-RA		chitin metabolic process	-1.19	0.011299025
CG6741-RA	Arc	adherens junction	-1.25	0.000232397
CG11905-RB		microtubule associated complex	-1.42	0.003637849
CG2198-RB	Amalgam	cell adhesion	-1.43	0.000436206
CG14534-RA		cuticle protein	-1.62	0.000705653
CG14957-RA		chitin binding protein; hormone induced ?	-1.89	0.000928449
CG7811-RA	Black	cuticle pigmentation	-2.00	0.004398815
CG10529-RA	Lcp65Ae	cuticle protein	-2.03	0.3175523
CG12063-RA		membrane matrix protein	-2.09	0.000233107
CG6955-RA	Lcp65Ad	cuticle protein	-2.12	0.001721654
CG8634-RA	Cpr65Ec	cuticule protein	-2.12	0.016934938
CG10297-RA	Acp65Aa	cuticle protein	-2.16	1.50E-05
CG5756-RB		chitin binding protein; Hox target?	-2.50	0.003778148
CG7076-RA	Cpr66Cb	cuticule protein	-2.86	0.000465549
CG14687-RA		myosin light chain binding	-2.88	0.000664679
CG33265-RA		chitin binding protein	-3.65	0.000255474
transport				
CG17137-RA	Porin2	mitochondrial transport	-1.02	0.103168312
CG7442-RA		secondary active organic cation transmembrane transporter	-1.03	0.000851822
CG5805-RA		transmembrane transporter activity	-1.04	0.002063842
CG31787-RA		transport	-1.05	0.038834906
CG11278-RA	Syntaxin 13	intracellular protein transport	-1.06	0.000450942
CG2969-RA	Atet	ATPase activity, transmembrane movement of substances	-1.09	0.000422922
CG8925-RA		carnitine transporter activity	-1.19	0.000670016
CG6331-RA	Organic cation transporter	organic cation transmembrane transporter activity	-1.23	0.000895287
CG12841-RA	Tetraspanin 42Ek	transmembrane transporter activity	-1.26	0.000456354
CG14855-RA		secondary active organic cation transmembrane transporter	-1.26	0.001188393
CG7777-RA		water transmembrane transporter activity	-1.27	0.000424974
CG2913-RA	Yin	oligopeptide transport	-1.33	0.009363689
CG1086-RB	Glucose transporter 1	glucose transmembrane transporter activity	-1.34	0.000146301
CG3327-RA	Early gene at 23	ATPase activity, transmembrane movement of substances	-1.38	0.000476913
CG17036-RA		reduced folate carrier activity	-1.39	0.000571786
CG13426-RA		protein transporter activity	-1.49	0.048888373
CG3994-RA		Zn transporter activity; detoxification	-1.92	9.18E-06
CG10440-RA		K+-channel	-2.11	2.84E-05
CG3823-RA		transporter activity; vitamin E binding	-2.24	0.000259571

Flybase ID	name	function	M-value	p-value
CG31636-RA		transporter activity; vitamin E binding	-2.25	3.26E-06
CG2663-RA		transporter activity; vitamin E binding	-2.26	2.47E-05
CG31634-RA	Oatp26F	organic anion transport	-2.37	0.006437775
CG1732-RA		Na-neurotransmitter symporter	-2.40	5.71E-05
CG17632-RA	Brown	ABC transporter	-2.62	0.000946577
CG17975-RA	Sugar transporter 2	glucose transmembrane transport	-2.65	1.17E-05
CG3649-RA		transmembrane transport (major facilitator)	-2.77	4.18E-05
CG6600-RA		major facilitator superfamily	-3.17	0.006253709
unknown function				
CG5780-RA		unknown function	-1.00	0.007533661
CG15525-RA		unknown function	-1.00	0.002540102
CG2837-RB		unknown function	-1.01	0.003386318
CG6685-RA		unknown function	-1.01	0.005627503
CG18249-RA		unknown function	-1.01	0.002901904
CG12674-RA		unknown function	-1.02	0.115112976
CG1124-RA		unknown function	-1.02	0.004859535
CG6900-RA		unknown function	-1.02	0.002040502
CG11882-RA		unknown function	-1.03	0.005511983
CG5968-RA		unknown function	-1.03	0.068830686
CG13962-RA		unknown function	-1.03	0.013747322
CG15210-RA		unknown function	-1.03	0.002801835
CG9686-RA		unknown function	-1.04	0.017348232
HDC06936		unknown function	-1.05	0.000909403
CG31600-RA		unknown function	-1.05	0.061005948
CG12880-RA		unknown function	-1.05	0.000475714
CG8678-RA		unknown function	-1.06	0.000896597
CG32597-RA	Lethal (1) G0469	unknown function	-1.06	0.007909145
CG6675-RA		unknown function	-1.06	0.058952897
CG8031-RA		unknown function	-1.06	0.000112519
CG3831-RA		unknown function	-1.06	8.44E-05
CG10332-RA		unknown function	-1.06	0.00993093
CG10933-RA		unknown function	-1.07	0.000313902
CG13905-RA		unknown function	-1.08	0.000523405
CG31447-RA		unknown function	-1.08	0.002018493
CG31901-RA	Mur29B	unknown function	-1.09	0.000155437
CT36057		unknown function	-1.09	3.54E-05
CG13618-RA		unknown function	-1.10	0.009970733
HDC06631		unknown function	-1.10	0.000287518
CT34146		unknown function	-1.11	0.006272368
CG32023-RA		unknown function	-1.12	0.018982104
LD44795		unknown function	-1.13	0.016051025
CG5812-RA	GCR(ich)	unknown function	-1.13	0.012382207
CG5741-RA		unknown function	-1.13	0.000215764

Flybase ID	name	function	M-value	p-value
CG6596-RA		unknown function	-1.13	0.043834632
CG1999-RA		unknown function	-1.13	0.065477215
CG31323-RA		unknown function	-1.13	0.002633596
CG13116-RA		unknown function	-1.14	0.000952807
CG31324-RA		unknown function	-1.14	0.001543399
CG5194-RA		unknown function	-1.14	0.022384661
CG10912-RA		unknown function	-1.16	0.000477485
CG33096-RA		unknown function	-1.17	0.001783652
CG11275-RA		unknown function	-1.17	8.01E-05
CG18343-RA		unknown function	-1.17	7.87E-06
CG9196-RA		unknown function	-1.17	2.33E-05
CG31538-RA		unknown function	-1.17	0.032547788
CG11474-RB		unknown function	-1.17	0.000241819
CG14567-RA		unknown function	-1.18	5.71E-05
CG9837-RA		unknown function	-1.18	0.00608643
CG11079-RA		unknown function	-1.18	7.19E-06
CG13255-RA		unknown function	-1.19	9.01E-05
CG3223-RA		unknown function	-1.19	0.000347439
CG32259-RB		unknown function	-1.19	0.047638928
CG31861-RA		unknown function	-1.21	0.078331135
CG13186-RA		unknown function	-1.21	0.024916429
CG14109-RA		unknown function	-1.21	0.000148046
CG4180-RB	Lethal (2) 35Bg	unknown function	-1.21	0.000125696
CG1273-RB		unknown function	-1.22	0.007381521
CG33252-RA		unknown function	-1.22	0.138094302
CG15917-RA		unknown function	-1.22	0.003755293
CG18190-RA		unknown function	-1.22	0.005409278
CG13488-RA		unknown function	-1.23	0.012692175
CG8160-RA		unknown function	-1.24	0.006389851
CG9801-RA		unknown function	-1.24	0.000151456
CG4438-RA		unknown function	-1.24	0.121741995
CG14275-RA		unknown function	-1.27	0.000408413
CG12655-RA		unknown function	-1.30	0.008200752
CG15544-RA		unknown function	-1.30	0.0013384
LP03188		unknown function	-1.30	0.00044247
CG15905-RA		unknown function	-1.31	0.002276054
CG32628-RA		unknown function	-1.32	3.27E-06
HDC12400		unknown function	-1.33	0.00588416
CG40294-RA		unknown function	-1.33	0.001679976
CG7841-RA		unknown function	-1.33	0.001312148
CG11737-RA		unknown function	-1.34	0.000100478
CG31525-RA		unknown function	-1.35	0.057378312
CG9040-RA		unknown function	-1.35	1.19E-05
CR_TC_GH03576		unknown function	-1.36	0.003533175

Flybase ID	name	function	M-value	p-value
CG12470-RA		unknown function	-1.37	0.045890788
CG6234-RA		unknown function	-1.37	0.000683634
CG2444-RA		unknown function	-1.38	0.000486507
CG1499-RA		unknown function	-1.42	0.002227539
CG15818-RA		unknown function	-1.42	0.000210766
CG30417-RA		unknown function	-1.43	0.029216772
HDC14735		unknown function	-1.43	0.001588422
CG32605-RA		unknown function	-1.45	0.031270511
CG15068-RA		unknown function	-1.46	0.014550876
RE54004		unknown function	-1.47	0.000467623
DM.2L.4959		unknown function	-1.47	0.073194939
CG7778-RA		unknown function	-1.49	0.000554144
CG2201-RA		unknown function	-1.52	9.05E-05
CG13856-RA		unknown function	-1.55	0.030714417
CG32115-RA		unknown function	-1.59	0.00058614
CG12481-RA		unknown function	-1.62	0.010590502
CG13024-RA		unknown function	-1.62	0.004604914
CG13082-RA		unknown function	-1.64	0.001833063
CG1172-RA		unknown function	-1.66	7.07E-06
CG2082-RE		unknown function	-1.67	0.003773799
CG15208-RA		unknown function	-1.70	0.069119011
CG33143-RC		unknown function	-1.72	4.70E-05
CG15756-RA		unknown function	-1.73	0.000435253
CG10516-RA		unknown function	-1.77	0.003092264
CG18622-RA		unknown function	-1.78	0.000183643
CG2082-RA		unknown function	-1.79	3.38E-07
CG2277-RA		unknown function	-1.88	5.26E-05
CG5391-RA		unknown function	-1.90	0.003789249
CG8483-RA		unknown function	-1.90	0.0001568
CG13722-RA		unknown function	-2.07	0.000618774
CG13314-RA		unknown function	-2.27	0.001880425
CG16836-RA		immune response	-2.27	0.001205338
CG16886-RA		unknown function	-2.29	0.0004613
CG34296-RA		unknown function	-2.92	5.79E-05
CG4151-RA		unknown function	-3.10	0.005671292
CR43242		unknown sequence	-3.37	9.84E-05
CR33948		unknown sequence	-3.54	0.000443585
CG5697-RA		unknown function	-4.47	0.006395644
CG9822-RA		unknown function	-4.79	0.003831544
CG42717		unknown function	-4.98	5.16E-05
