# Supplementary material for: CHD1 Contributes to Intestinal Resistance against Infection by P. aeruginosa in Drosophila melanogaster
Source: PLoS One. 2012 Aug 13;7(8):e43144. doi: 10.1371/journal.pone.0043144 (PMC3418260; doi:10.1371/journal.pone.0043144)
Supplement: Table S3 — PCR Primer sequences. (PDF) [file pone.0043144.s009.pdf]

**primer**	**sequence [5´- 3`]**
RT-qPCR AMPs & negative Imd regulators	
Rpl32 fw	CAACATCGGTTACGGATCGA
Rpl32 rev	AATCCGGTGGGCAGCAT
AttC fw	GATCGCAACACAAGGCTAGA
AttC rev	GAAGGGTCCACTTGTCCACT
DipB fw	CCAAAGCAAGGATTCGATCT
DipB rev	AAGGAGTGGCGTCCATTG
Metch fw	GTGCTGGCAGAGCCTCATC
Metch rev	GCGACGGCCTCGTATCG
dro2 fw	GCCGCCAATATGGCTGAT
dro2 rev	GCAGGGACCCTTGTATTTGC
dro3 fw	CTGGAACTTTCGGAGGTCCTT
dro3 rev	AGAGACGGCGGCACTTTTC
PGRP_SC2 fw	CCACACCGCTGGAAACTACTG
PGRP_SC2 rev	TTCTGCAGCTGTGTGATGCA
PGRP_SB1 fw	CCCCATCCGCCCAGAT
PGRP_SB1 rev	TGCTTGGCCAGCTCGATTA
PGRP_LB fw	AGCGTGACTTAATTTCGCACA
PGRP_LB rev	TATGTTGACGATGCCGAAGAG
PGRP_LC fw	TCCAATCGAAATCGGAAGAG
PGRP_LC rev	GGCGAAGATGTCTTTCCAAC
caudal fw	CAAGGAGCGCAAGCAGAAC
caudal rev	CCCACGCCCATCACGTT
pirk fw	CAACTGCAAGTAATCGGCAA
pirk rev	AGATCCTTCCGTCGTTACCC
caspar fw	GAAACTTTGCAAGCCGACAT
caspar rev	GCGTCTAGCATCTTCTTCGG
dUSP36 fw	TGTGGCACGTGCTTAAACTGTC
dUSP 36 rev	GCTGATGATTGTGGTGGTTG

**primer**	**sequence [5´- 3`]**
RT-qPCR bacterial load	
16S rRNA fw	CCATGAAGTCGGAATCGCTAG
16S rRNA rev	ACTCCCATGGTGTGACGG
GB_EW/707 fw	AGAGATGGGCATTTCTTC
GB_EW/707 rev	AGAGATCAGCATGATGTC
AB_EW911 fw	CATGCAAGTCGCACGGA
AB_EW911 rev	TTCCTCCACAAGCGGATC
PA23FP fw	TCCAAGTTTAAGGTGGTAGGCTG
PA23FP rev	CTTTTCTTGGAAGCATGGCATC
